# Improvement of Free Fatty Acid Secretory Productivity in *Aspergillus oryzae* by Comprehensive Analysis on Time-Series Gene Expression

**DOI:** 10.3389/fmicb.2021.605095

**Published:** 2021-04-09

**Authors:** Pui Shan Wong, Koichi Tamano, Sachiyo Aburatani

**Affiliations:** ^1^Cellular and Molecular Biotechnology Research Institute, National Institute of Advanced Industrial Science and Technology (AIST), Tokyo, Japan; ^2^Bioproduction Research Institute, National Institute of Advanced Industrial Science and Technology (AIST), Sapporo, Japan; ^3^Computational Bio Big-Data Open Innovation Laboratory (CBBD-OIL), National Institute of Advanced Industrial Science and Technology (AIST), Tokyo, Japan

**Keywords:** *Aspergillus oryzae*, free fatty acid, secretory productivity, acyl-CoA synthetase mutant, Triton X-100, RNA-seq, time-series gene expression

## Abstract

*Aspergillus oryzae* is a filamentous fungus that has historically been utilized in the fermentation of food products. In recent times, it has also been introduced as a component in the industrial biosynthesis of consumable compounds, including free fatty acids (FFAs), which are valuable and versatile products that can be utilized as feedstocks in the production of other commodities, such as pharmaceuticals and dietary supplements. To improve the FFA secretory productivity of *A. oryzae* in the presence of Triton X-100, we analyzed the gene expression of a wild-type control strain and a disruptant strain of an acyl-CoA synthetase gene, *faaA*, in a time-series experiment. We employed a comprehensive analysis strategy using the baySeq, DESeq2, and edgeR algorithms to clarify the vital pathways for FFA secretory productivity and select genes for gene modification. We found that the transport and metabolism of inorganic ions are crucial in the initial stages of FFA production and revealed 16 candidate genes to be modified in conjunction with the *faaA* disruption. These genes were verified through the construction of overexpression strains, and showed that the manipulation of reactions closer to the FFA biosynthesis step led to a higher increase in FFA secretory productivity. This resulted in the most successful overexpression strains to have an FFA secretory productivity more than two folds higher than that of the original *faaA* disruptant. Our study provides guidance for further gene modification for FFA biosynthesis in *A. oryzae* and for enhancing the productivity of other metabolites in other microorganisms through metabolic engineering.

## Introduction

Free fatty acids (FFAs) are lipid compounds that have a carboxyl group in a free form within the molecule. FFAs are valuable, because their derivatives can be used as source materials for biofuels, various pharmaceuticals, and dietary supplements (Lennen and Pfleger, 2013; Tamano, 2014). For example, FFAs can be chemically combined with methanol in an acidic environment to generate fatty acid methyl esters (FAMEs) that are generally used as biodiesel fuel (Ichihara and Fukubayashi, 2010). In particular, FAMEs derived from biologically produced fatty acid precursors have been used as additives in petroleum-derived diesel fuel (Peralta-Yahya et al., 2012). FFAs can also act as feedstocks for the production of pharmaceutical compounds [e.g., prostaglandin E1 (PGE_1_)] and functional lipids [e.g., eicosapentaenoic acid (EPA) and docosahexaenoic acid (DHA)] (Lennen and Pfleger, 2013; Zhou et al., 2016). PGE_1_ inhibits platelet aggregation and vasodilation and has thus been used as a pharmaceutical for patients with ischemic or myocardial reperfusion injury (Zhu et al., 2017). EPA and DHA are possibly beneficial in the normal aging processes of the brain and are thus used as dietary supplements (Dyall, 2015).

Microbial free fatty acid production has been researched in the bacterium *Escherichia coli* (Youngquist et al., 2013; Cao et al., 2014; Wu et al., 2014; Shin et al., 2016; Li et al., 2018), the yeast *Saccharomyces cerevisiae* (Chen et al., 2014; Leber et al., 2015), the filamentous fungus *Aspergillus oryzae* (Tamano et al., 2013, 2015; Tamano and Miura, 2016), and photosynthetic microorganisms, such as microalgae (Ruffing, 2013, 2014; Eungrasamee et al., 2020).

Among them, *A. oryzae* is a food-safe filamentous fungus that has been used in the production of fermented foods, such as rice wine (*sake*), soy sauce (*shoyu*), and soybean paste (*miso*), since ancient times, in Japan, where its characteristic fermentation processes were used for converting starch, proteins, and lipids into a range of small molecules, such as glucose, amino acids, and fatty acids (Abe et al., 2006), to give the fermented foods an enhanced flavor. More recently, the metabolic pathways that drive fermentation in *A. oryzae* have been directed to meet the requirements of the industrial-scale production of compounds that have industrial applications, such as amylase, protease, and lipase (Goldman and Osmani, 2007). Even though producing valuable compounds and enzymes using other microorganisms has been attempted, *A. oryzae* has an advantage in this industry. Owing to its long history of use in the fermentation of food products, the compounds produced by *A. oryzae* are generally regarded as safe for human consumption. In this study, we attempted to improve the secretory productivity of FFAs in *A. oryzae*, as this organism already possesses the ability to produce these compounds.

Typically, FFAs are produced via glycolysis, tricarboxylic acid (TCA) cycle, and lipid biosynthesis pathways, such as by fatty acid synthase (FAS). However, FFAs also act as intermediates in the recycling of lipids by degradation and are subjected to acylation by acyl-CoA synthetase and degradation by subsequent beta-oxidation. Therefore, to control the amount of FFA being produced, the function and expression of all genes involved in the relevant metabolic pathways are being investigated using the *A. oryzae* RIB40 genome as the representative genome of the wild-type strain (Machida et al., 2005). Initial verified and predicted gene annotations revealed the FAS gene (Tamano et al., 2013) and the acyl-CoA synthetase gene (Tamano et al., 2015) (*faaA*) in *A. oryzae*. Overexpression of FAS resulted in a 2.1-fold increase in FFA productivity, and the disruption of *faaA* resulted in a 9.2-fold increase in FFA productivity. Combined FAS overexpression and *faaA* disruption did not result in an increase in FFA productivity above that achieved by *faaA* disruption (Δ*faaA* strain) alone and subsequent research has thus prioritized the development of FFA production in the Δ*faaA* strain.

Because the Δ*faaA* strain is considered to contain no acyl-CoA synthetase activity responsible for the degradation of FFA to fatty acyl-CoA (Tamano et al., 2015), any further enhancement of FFA production needs to support and complement the changes brought about by the disruption of *faaA*. Absence of the FFA degradation process results in dysfunctional beta-oxidation metabolic activity, as fatty acyl-CoA is a beta-oxidation initiator and leads to drastic increases in FFA production in wild-type strains. Additionally, FFA production is confirmed to start in the logarithmic growth phase, specifically in the second day of a 5-day culture period (Tamano et al., 2013). Therefore, the secretion of accumulated FFA was examined to provide more intracellular space to accommodate continued FFA production that would lead to further improvement in the overall FFA yield from the Δ*faaA* strain. As a result, when a non-ionic surfactant, Triton X-100, was supplemented to a liquid culture of the Δ*faaA* strain, it was found that Triton X-100 caused a spontaneous extracellular release of FFA into the culture medium at more than 80% efficiency without causing any growth inhibitory effects (Tamano et al., 2017). The mechanism by which this occurs is deduced to be related to an increase in membrane permeability which causes the secretion of FFA by diffusion. Although this combination of genetic modification and Triton X-100 supplementation increased the extracellular release of FFA, it did not significantly increase FFA yield. Instead, it resulted in a modest increase of approximately 1.1 folds that was insufficient for large-scale production. Thus, further investigation was necessary to uncover the unknown mechanisms that restricted the combined effects of the *faaA* disruption and Triton X-100 supplementation and to remove them to further improve FFA yield.

Here, we devised and implemented a comprehensive methodology that utilized several differential expression algorithms to uncover the unknown mechanisms obstructing the *faaA* disruption effect to further increase FFA secretory productivity. Because we anticipated the effect of the *faaA* disruption to vary with time, we applied our methodology to RNA-seq data obtained in the presence of Triton X-100 over a duration of 120 h. We started a cursory search with a selection of differential expression algorithms (Robinson et al., 2009; Hardcastle and Kelly, 2010; McCarthy et al., 2012; Love et al., 2014) and then examined the biological functions of the selected genes to identify the processes obstructing FFA productivity. We obtained an initial list of differentially expressed genes (DEGs), which were associated with FFA metabolism, and then, in combination with a list of manually curated genes, used it to isolate the candidate genes for modification. Finally, we verified those genes by overexpressing each of them in the Δ*faaA* strain to successfully show an increase in FFA secretory productivity by metabolic engineering.

## Materials and Methods

### Fungal Strains and Culture

We used an *A. oryzae* wild-type RIB40 strain distributed by the National Research Institute of Brewing (NRIB, Hiroshima, Japan) and its derivative *faaA* disruptant strain named Δ*faaA* (RIB40 Δ*ligD:ptrA* Δ*niaD:niaD* of *Aspergillus nidulans* Δ*pyrG:sC* of *A. nidulans* Δ*faaA:pyrG of A. nidulans*) or the uracil-auxotrophic Δ*faaA* strain named Δ*faaA_pyrG-* (RIB40 Δ*ligD:ptrA*Δ*niaD:niaD* of *A. nidulans*Δ*pyrG:sC* of *A. nidulans* Δ*faaA*). Both Δ*faaA* and Δ*faaA_pyrG-* strains were constructed in our previous study (Tamano et al., 2015; Tamano and Miura, 2016). We also used an *A. nidulans* wild-type A851 strain distributed by the Fungal Genetics Stock Center (Kansas City, MO, United States) in the preparation of DNA fragments of both an orotidine-5′-phosphate decarboxylase gene, named *AnpyrG*, as a uracil-auxotrophic selectable marker, and a promoter of the constitutive gene *Antef1*, named *AnPtef1*. Each strain was maintained on Czapek-Dox (CD) agar medium with or without 5 mM uridine and 10 mM uracil at 30°C (Tamano et al., 2007). The CD agar medium contained 2% glucose, and the glucose concentration was increased to 10% for the CD liquid medium. Cultures were prepared by inoculating 2.5 × 10^7^ spores of each *A. oryzae* strain in 50 mL of CD liquid medium supplemented with 1% (w/v) Triton X-100 (Code No. 648466, Merck Millipore, Billerica, MA, United States) in a 250 mL flask, followed by incubation at 30°C with shaking at 200 rpm for 48, 72, 96, 120, 144, or 168 h.

### RNA Isolation

Hyphae harvested after liquid culture were washed in 100 mL of autoclaved Milli-Q water and ground using a mortar and a pestle in liquid nitrogen. Frozen powders of ground hyphae were transferred to 10 mL of the ISOGEN reagent (Nippon Gene, Toyama, Japan), and total RNA was then extracted using a spin column attached to the ISOGEN, according to the manufacturer’s manual. Contaminating chromosomal DNA was removed from total RNA by treatment with RNase-free DNase I (New England Biolabs, Ipswich, MA, United States). DNase I-treated total RNA was then purified with an RNeasy^®^ mini kit (QIAGEN, Hilden, Germany), following the manufacturer’s instructions. RNA was eluted with 100 μL of nuclease-free water and stored at −80°C.

### RNA Sequencing

RNA samples were subjected to RNA sequencing (RNA-seq) in an Illumina HiSeq 2500, and the sequence reads were trimmed using cutadapt (Martin, 2011) and trimmomatic (Bolger et al., 2014). Reads were mapped using TopHat (Trapnell et al., 2009), Bowtie 1 (Langmead et al., 2009), and the *A. oryzae* RIB40 ASM18445v3 reference genome (BioProject: PRJNA20809). The expression data are publicly available at GenBank^1^ of the National Center for Biotechnology Information (accession number: PRJDB8293).

### Differential Gene Expression Analysis

**FIGURE 1:**
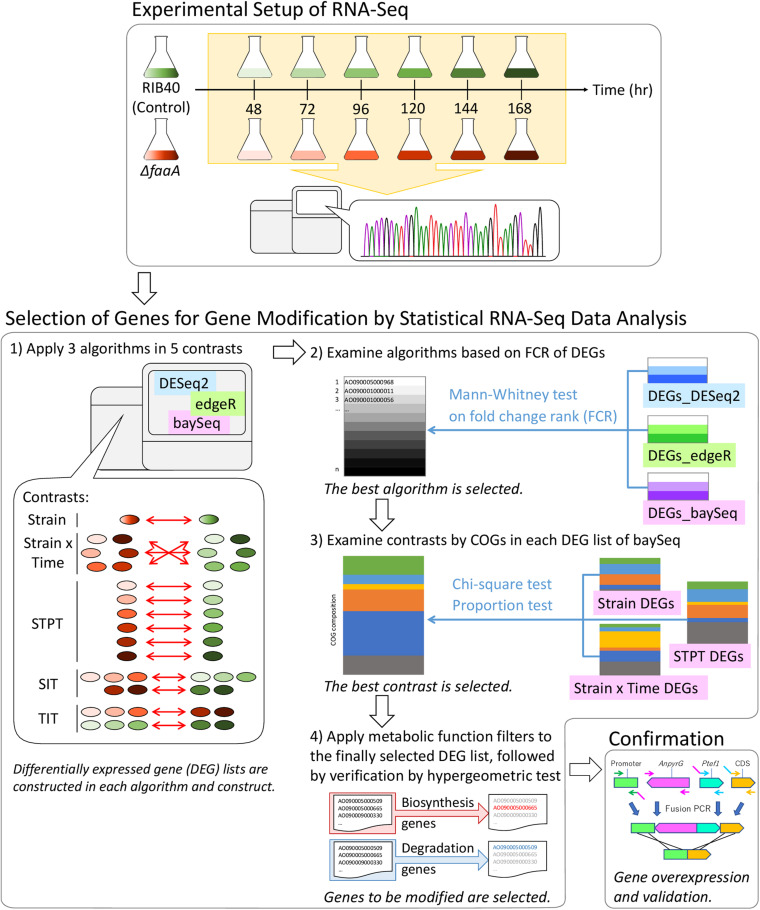
Flow chart of the methodology used to select candidate genes for genetic modification for increasing FFA secretory productivity. Genome-scale RNA samples were prepared in 24 h intervals, from 48 to 168 h, from the RIB40 control and Δ*faaA* strains. RNA samples were subjected to RNA-Seq and processed into normalized gene expression data for analysis. Analysis started with the extraction of differentially expressed genes by three algorithms (DESeq2, edgeR, and baySeq), followed by their comparison after being separated into five contrast categories: Strain, Strain × Time, Single Time Point (STPT), Strain Impact (SIT), and Time Impact (TIT). The resulting gene lists were analyzed by the Mann–Whitney test, and the results from the most robust method were further analyzed using clusters of orthologous groups (COG). Finally, the results were refined using functional gene lists (Supplementary Table 1) to reduce the number of final candidates that were then verified by genetic modification.

A flow chart of the multi-step comprehensive data analysis performed in this study is shown in Figure 1. In preparation for the analysis, the RNA-seq data were normalized and tested for differential expression with the baySeq (Hardcastle and Kelly, 2010), DESeq2 (Love et al., 2014), and edgeR (Robinson et al., 2009; McCarthy et al., 2012) algorithms implemented with their respective Bioconductor packages in the R statistical programming environment (R Core Team, 2019) and the TCC library (Sun et al., 2013). Genes with zero counts across all samples were removed before the data were normalized. The normalization method for contrasts with pseudoreplicates was trimmed mean of *M*-values (TMM), and the normalization method for contrasts without pseudoreplicates was DESeq2’s size factor estimation. The DEGs were selected with a false discovery rate (FDR) threshold of 0.05 for all algorithms, and a sample size of 10,000 iterations was used in the baySeq tests. The normalized data were comparatively analyzed using each algorithm described in section “Contrast Categories Applied to the RNA-Seq Data Analysis.”

#### Contrast Categories Applied to the RNA-Seq Data Analysis

The following five contrasts were used to isolate the sources of variation in the differential expression between strains.

##### Strain

Comparison between the RIB40 and Δ*faaA* strains using time point data as pseudoreplicates to identify prevailing genes affected by *faaA* disruption.

##### Strain × Time

Comparison between the RIB40 and Δ*faaA* strains with the time variable to identify genes affected by *faaA* disruption with an interaction term for strain and time.

##### Single time point (STPT)

Individual comparisons between the RIB40 and Δ*faaA* strains at 48, 72, 96, 120, 144, and 168 h to identify genes affected by the *faaA* disruption at specific time points.

##### Strain impact (SIT)

Comparisons between the RIB40 and Δ*faaA* strains in the early (48–96 h) and late (144–168 h) intervals, with the samples within an interval acting as pseudoreplicates, to identify genes affected by the *faaA* disruption during the logarithmic and stationary growth phases. The DEGs selected by these comparisons in the early and late intervals were referred to as S(early) and S(late), respectively.

##### Time impact (TIT)

Comparisons between early (48–96 h) and late (144–168 h) intervals for the RIB40 and Δ*faaA* strains, such that the samples within an interval acted as pseudoreplicates, to identify genes affected by chronological changes in both strains. The DEGs selected by these comparisons were referred to as T(control) and T(*faaA*) for the RIB40 and Δ*faaA* strains, respectively.

#### Examination of Algorithm Performance

The lists of DEGs from each algorithm and contrast (Strain, Strain × Time, STPT, SIT, and TIT) were compared using Mann–Whitney tests, based on their fold-change rank. Because the fold-change ranks of the DEGs were expected to be higher than average, one-sided Mann–Whitney test was used, with a *p*-value threshold of 0.05, and FDR was used to adjust for multiple testing.

#### Examination of Contrasts in baySeq Results

To confirm that there were functional differences between the baySeq results and the full data set and between the results themselves, the ratios of clusters of orthologous groups (COG) of proteins was inspected in each gene list. A COG ratio was the number of genes with a certain COG divided by the total number of genes with COG annotations. The COG ratios were verified by Chi-square test for goodness of fit, proportion test, and hypergeometric test. The COG annotations for *A. oryzae* were downloaded from the Comprehensive *Aspergillus oryzae* Genome Database^2^. Combination COGs were created for genes annotated with multiple COGs by concatenating the COG codes and separating them by “|”. COGs annotated in fewer than 15 genes in the full data set were merged into an “Other” combination COG, and genes without COG were labeled “NC.” COG “S” corresponds to “function unknown.”

The Chi-square goodness of fit test was performed first to compare the observed number of COGs in a DEG list with the expected number found in the full data set using a *p*-value threshold of 0.05 and FDR adjustment for multiple testing. As the DEG lists from the Strain, Strain × Time, SIT, and TIT tests were small, COGs with counts fewer than five were removed. In addition, proportion tests were carried out to test for differences in COG ratios between a DEG list and the full data set. In an effort to select target genes for modification, the results from the baySeq, Chi-square, and proportion tests were first used to determine a list of DEGs that were affected by the *faaA* disruption. Then, the FFA biosynthesis and degradation gene lists were used to selectively filter DEGs affected by *faaA* disruption from those that were specific to FFA metabolism. The filtered DEGs were then tested using one-sided hypergeometric tests to confirm that the number of filtered DEGs was greater than the number of FFA genes in the full data set.

### Manual Curation of Fatty Acid Biosynthesis or Degradation Gene Lists in *A. oryzae*

Lists of *A. oryzae* genes considered to play a role in FFA biosynthesis or degradation were manually identified via their COG annotations (Machida et al., 2005), followed by further reference to improved annotation (Vongsangnak et al., 2008) and functional analysis (Tamano et al., 2013, 2015) literature. Initially, 516 genes were selected using version 9 of an *A. oryzae* COG annotation list. The 516 genes were refined to 38 fatty acid synthesis genes and 25 fatty acid degradation genes by consulting with the Aspergillus Genome Database (AspGD). The lists are available in the Supplementary Materials (Supplementary Table 1).

### Overexpression of Individual Genes in the *A. oryzae faaA* Disruptant

Introduction of individual genes for overexpression into the Δ*faaA* strain was performed by replacing its native promoter with the *A. oryzae tef1* (AO090120000080) promoter named *Ptef1* or the *A. nidulans tef1* (AN4218) promoter named *AnPtef1*. The *Ptef1* and *AnPtef1* promoters have constitutively high transcriptional activities and have also been used in the overexpression of other genes in *A. oryzae* (Kitamoto et al., 1998). The primers used in this study are listed in Supplementary Table 2. Each overexpressed Δ*faaA* strain was constructed using the following steps. The Δ*faaA_pyrG-* strain (Tamano and Miura, 2016) was transformed with the DNA fragment of each gene to be overexpressed. Two 1-kb DNA fragments of the promoter and the coding region, starting from the start codon of each gene, were amplified with LU/LL and RU/RL primer pairs from the *A. oryzae* RIB40 genomic DNA template (Supplementary Figures 1A–N). These DNA fragments were mixed with a 1.8-kb fragment of *AnpyrG*, and a 1-kb *Ptef1* or *AnPtef1* DNA fragment and were then subjected to fusion PCR with primers LU and RL and an extension time of 5–6 min (1 min/kb). The resultant fragment harboring a portion of each gene with the promoter replaced with *Ptef1* or *AnPtef1* was introduced into the Δ*faaA_pyrG-* strain by transformation. The transformants selected on CD agar medium were subjected to a clone check by PCR with the LU (or cU) and LL (or cL) primers.

### Quantification of Extracellular FFA

For the quantification of extracellular FFAs with the Free Fatty Acids Half-micro Test Kit (Roche Applied Science, Mannheim, Germany) following the manufacturer’s protocol, 5 μL of each culture supernatant was used. In detail, the secretory productivity was calculated by dividing the FFA concentration in the culture supernatant by the dry cell weight, which was obtained by separation of hyphae from the culture supernatant with Miracloth (EMD Chemicals, San Diego, CA, United States) followed by washing and lyophilization. The secretory production yield of FFAs was calculated by converting the FFA concentration in the culture supernatant to equivalent weight of free PA per liter of culture medium. The quantity of FFAs from the overexpressed Δ*faaA* strains was compared to that from the Δ*faaA* strain using Student’s *t*-test. Strains were considered to have a significant difference in FFA quantity compared to that of the Δ*faaA* strain when then *p*-value was <0.01.

### Composition Analysis of Extracellular FFAs

To analyze the composition of extracellular FFAs, 1 mL of *A. oryzae* culture supernatant was transferred to a 2 mL screw-cap microtube. After 1 mL of chloroform was added, the mixture was agitated by vigorous shaking. After centrifugation at 13,000 × *g* at room temperature for 10 min, the upper aqueous layer was removed with a 1 mL tip. Then, a 450 μL aliquot of the lower chloroform fraction was transferred to a 10 mL screw-cap glass centrifuge tube. The transferred chloroform fractions containing the intracellular lipids were evaporated in a centrifugal vacuum concentrator. The resulting precipitates were dissolved in 300 μL of ethanol. The FFA molecules in each lipid sample were specifically labeled with 2-nitrophenylhydrazine at the carboxyl group using an FFA analysis kit from YMC (Kyoto, Japan). After the reaction was completed, the labeled FFAs were extracted with 5 mL of hexane, and 2 mL of the hexane fraction was evaporated in a centrifugal vacuum concentrator. The labeled FFA precipitates were dissolved in 300 μL of methanol and stored at 4°C prior to measurement by high performance liquid chromatography (HPLC) using Chromaster (Hitachi, Ibaraki, Japan). Similarly to the aforementioned samples, 100 μL of each FFA standard solution at three concentrations (1.67, 3.33, and 5 mM) dissolved in ethanol was used to construct standard curves with linear regression. Four FFA standards (PA, SA, OA, and LA) were used. The labeling reaction and successive treatments were performed using the same protocols as those used for sample preparation. Ten microliters of each labeled FFA sample or standard dissolved in methanol was applied to HPLC equipped with a YMC-Pack FA column (YMC; 0.46 × 25 cm) using an acetonitrile-water (85:15) mixture as the mobile phase at a flow rate of 0.7 mL/min and a column temperature of 35°C. Labeled FFAs were detected by monitoring absorbance at 400 nm. FFA composition and quantity for each sample were calculated based on the peak area using the corresponding labeled FFA standard curves.

## Results

### Certification of Algorithms for DEG Identification

RNA-Seq expression data were obtained from the *A. oryzae* RIB40 control strain and the Δ*faaA* strain cultured in the presence of 1% Triton X-100 and sampled at 48, 72, 96, 120, 144, and 168 h, as described in section “Materials and Methods.” Data were processed from 12,682 predicted and transcribed genes to select 12,137 genes after removing genes with no sequence reads. Three differential expression algorithms were used to identify the genes affected by the *faaA* disruption so that FFA secretory productivity could be increased by gene modification: baySeq (Hardcastle and Kelly, 2010), DESeq2 (Love et al., 2014), and edgeR (Robinson et al., 2009; McCarthy et al., 2012; Figure 1). These algorithms were applied to five contrast categories, and the DEG lists were then compared to identify the algorithm with the most robust performance (Supplementary Table 3).

To compile the list of candidate genes for modification, the most robust algorithm was determined by comparing the fold-change ranks between the DEG lists and the full data set. This was performed by Mann–Whitney test, and nine baySeq DEG lists were found to contain genes of significantly higher fold-change ranks than a randomly sampled list of the same size (*p*-value < 0.05). In comparison, the lists from edgeR and DESeq2 were only significant for six and one list, respectively (Supplementary Table 4). Because the majority of baySeq DEG lists contained genes with a high fold change, they were used in the subsequent steps of selecting genes for modification.

### Validating the baySeq Results for Bottleneck Gene Selection

To confirm that the differential expression in the baySeq results was associated with the *faaA* disruption and to select genes for modification, the DEGs within each DEG list were investigated using COG functional gene annotation. First, all the genes in the full data set were annotated with COGs. There were initially 132 unique COGs that were reduced to 35 for clarity. This was done by introducing the “NC” and “Other” combination COG terms for genes with no COG and genes with rare combinations of COGs, respectively. COGs were annotated in a large number of genes, ranging from 8,492 genes annotated with “NC” and 15 genes annotated with “J|K|L|R.” The top ten annotated COGs were single-term COGs (Supplementary Table 5). Some of the absent COGs were “N” (cell motility), “W” (extracellular structures), “Y” (nuclear structure), “A” (RNA processing and modification), and “B” (chromatin structure and dynamics), as expected for FFA accumulation. The COGs in each baySeq DEG list were used to calculate COG ratios to represent their respective list. The COG ratios were then compared between DEG lists using the Chi-square test for goodness of fit, proportion test, and hypergeometric test.

To confirm that the observed COG counts in the DEG lists were significantly shifted toward specific metabolic activity compared to the expected counts in the full data set, the COG counts between the baySeq DEG lists and the full data set were compared using the Chi-square goodness of fit test. The Chi-square test showed significant differences in COG counts except in the S(late) DEG list (Supplementary Table 6). This strongly indicates an absence of any particular shift in metabolic activity in the 144–168 h period.

**FIGURE 2:**
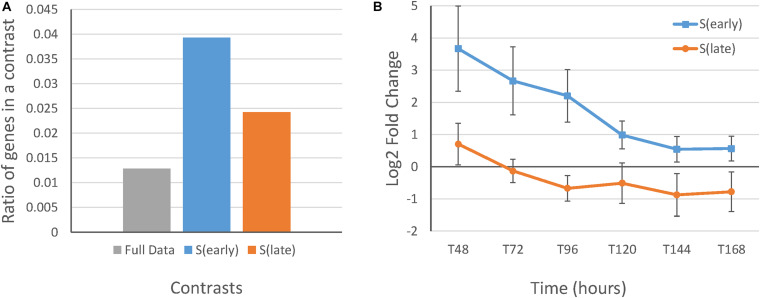
Gene expression and ratios of differentially expressed genes from the S(early) and S(late) results with the COG function “P” compared to those in the full data set. **(A)** Ratio of genes with the COG “P” (inorganic ion transport and metabolism) in the full data set and the DEG lists of S(early) and S(late). **(B)** Average log fold-change between the RIB40 control and Δ*faaA* strains in the DEG lists of S(early) and S(late) with the COG “P” with standard error bars. There were nine genes in the S(early) results and ten genes in the S(late) results (Supplementary Table 9).

Subsequently, proportion tests were performed to test for significant differences in COG ratios between the baySeq DEG lists in the STPT, SIT, and TIT contrast categories. This was followed by a test for significant differences in COG ratios between the DEG lists of S(early) and S(late), and T(control) and T(faaA) separately, which confirmed that the contrasts isolated the *faaA* disruption effect on strain and time (Supplementary Table 7). First, there was a significantly higher ratio for “P” (inorganic ion transport and metabolism) in the DEG lists of the SIT contrast category than in the full data set. The ratio for “P” was also significantly higher in the DEG lists of S(early) than those of S(late) (Supplementary Table 8). Further, the average fold change between the RIB40 control strain and the Δ*faaA* strain in genes annotated with “P” in the SIT DEG lists concurred with the COG ratio results. These results showed that the genes annotated with “P” in the DEG list of S(early) were more highly expressed than those in the DEG list of S(late) (Figure 2). This observed decrease in the *faaA* disruption effect in “P” may be the reason for the lack of overlap between S(early) and S(late) (Supplementary Table 9). In terms of theorized gene ontologies (GO), the DEG list of S(early) containing “P” included three genes related to oxidation-reduction process and three genes related to cation transport, whereas the DEG list of S(late) with COG “P” included genes related to processes, such as sulfite, copper ion, phosphate ion transport, hyphal and filamentous growth, and cellular response to biotic and abiotic stimulus. In contrast, there were no significantly different COG ratios between the DEG lists of T(control) and T(faaA). Overall, these results reveal a strong *faaA* disruption effect until 120 h, when the increased FFA production initiated a metabolic change that aligned gene expression in the *faaA* disruptant to be more similar to that in the control strain. These findings confirm that 120 h is a suitable divider between the early and late stages of FFA production.

Similarly, the COG ratios in the DEG lists of the STPT contrast category were analyzed with proportion tests. When those COG ratios were compared to those in the full data set, the COG ratios were very different for the COGs “J” (translation, ribosomal structure, and biogenesis), “O” (posttranslational modification, protein turnover, chaperones), and “NC” (Supplementary Table 7). However, no difference was observed in COG ratios between time points.

As the proportion tests verified that the COG ratios were similar among all DEG lists in the STPT contrast category, the lists were considered combinable by intersection for the subsequent selection of genes for modification. In addition to the proportion test results, the DEG lists contained genes with high fold changes between the RIB40 and Δ*faaA* strains in general, and their COG ratios were significantly different from those of the full data set. Collectively, these findings suggested the intersection of the DEG lists in the STPT contrast category to be the most appropriate for selecting genes for modification.

### Bottleneck Gene Selection and Verification for Increasing FFA Secretory Productivity in the *A. oryzae faaA* Disruptant

To confirm increased FFA secretory productivity by gene modification, genes were selected by a strict criterion. As described above, the initial group of candidates was selected by the intersection of the DEG lists in the STPT contrast category and contained 1,450 genes (Supplementary Table 3). These genes were then reduced to those suspected of being associated with FFA metabolism using the manually curated biological knowledge of gene functions in the FFA biosynthesis and degradation lists (Supplementary Table 1). The resulting list of candidates contained 16 biosynthesis genes (Table 1) and nine degradation genes. These genes were verified for representation of biosynthesis and degradation genes by comparison to the full data set using hypergeometric tests (Supplementary Table 10). Because additional gene disruption in conjunction with *faaA* disruption was deemed unnecessary, only the overexpression of biosynthesis genes was performed.

**TABLE 1:** The FFA biosynthesis genes that were selected for gene modification.

Gene ID	BLASTP NR	Annotation
AO090023000205	Probable ATP citrate lyase subunit 2 (*Neurospora crassa*)	ATP citrate lyase
AO090023000206	Hypothetical protein (*Neurospora crassa*)	ATP-citrate lyase
AO090011000838	Acetyl-CoA carboxylase (EC 6.4.1.2) – *Emericella nidulans*	Acetyl-CoA carboxylase
AO090124000083	Fatty acid synthase, alpha subunit (*Emericella nidulans*)	3-oxoacyl-[acyl-carrier-protein] reductase
AO090124000084	Fatty acid synthase, beta subunit (*Emericella nidulans*)	Enoyl reductase domain of yeast-type FAS1
AO090012000721	Hypothetical protein (*Neurospora crassa*)	Palmitoyl protein thioesterase
AO090005001021	Long chain polyunsaturated fatty acid elongation enzyme (*Isochrysis galbana*)	Long chain fatty acid Elongase
AO090102000393	Hypothetical protein (*Neurospora crassa*)	Fatty acyl-CoA Elongase
AO090026000492	Microsomal beta-keto-reductase; Ybr159wp (*Saccharomyces cerevisiae*)	17 beta-hydroxysteroid dehydrogenase III
AO090005000456	Stearic acid desaturase (*Emericella nidulans*)	Stearic acid desaturase
AO090023000893	Hypothetical protein (*Neurospora crassa*)	Fatty acid desaturase
AO090011000488	Unnamed protein product (*Podospora anserina*)	Delta 6-fatty acid desaturase
AO090001000224	Oleate delta-12 desaturase (*Aspergillus flavus*)	Oleate delta-12 desaturase
AO090102000339	Fatty acid desaturase (*Aspergillus oryzae*)	
AO090011000863	Hypothetical protein (*Neurospora crassa*)	Diacylglycerol O-acyltransferase
AO090701000644	Lipase (*Aspergillus parasiticus*)	Diacylglycerol lipase

*Using the Chi-square test, proportion test, and hypergeometric test along with metabolic function filters, the 1,450 candidates, which were differentially expressed between the RIB40 control and Δ*faaA* strains in the intersection of the DEG lists of the STPT contrast category from the baySeq results, were narrowed down to 16 genes for gene overexpression.*

**FIGURE 3:**
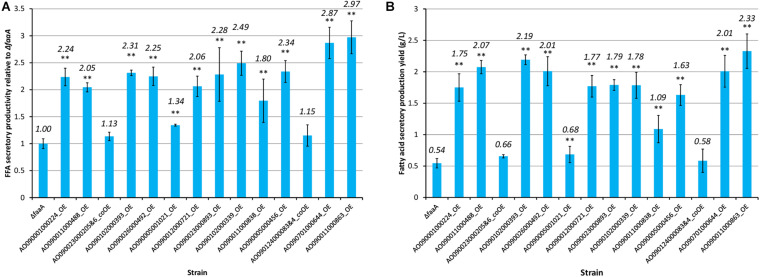
FFA secretory productivity and yield of the 16 overexpression mutants relative to those of the Δ*faaA* strain. Each gene overexpression mutant derived from the Δ*faaA* strain was tested for both FFA secretory productivity **(A)** and FFA secretory production yield **(B)** in conjunction with the Δ*faaA* strain. The overexpression mutants containing two overexpressed genes were named with the two gene IDs along with “&.” This also indicated that they are subunits of the same enzyme complex and were modified together. The productivities of the 16 overexpressed mutants and the parental Δ*faaA* strain were tested using Student’s *t*-test and those with *p*-value < 0.01 are marked with “**”. The actual yield of each mutant is shown above each bar in italics.

**FIGURE 4:**
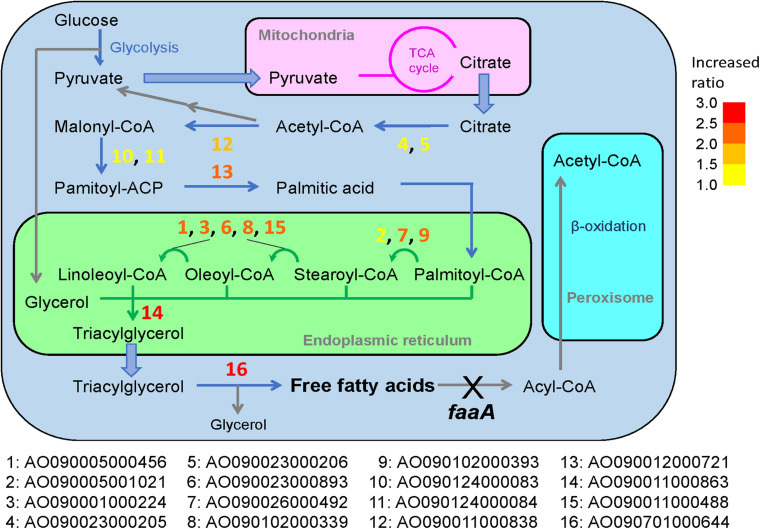
Predicted reactions of the overexpressed genes in the general metabolic pathway for FFA production in the Δ*faaA* strain. The 16 genes that were subjected to overexpression modification in addition to *faaA* disruption were designated with numbers from 1 to 16. These numbers are overlaid on the metabolic pathway map and marked by a corresponding color to indicate the FFA secretory productivity change as a ratio compared to the Δ*faaA* strain. Increasing FFA secretory productivity is shown by the color of the gene number, shifting from yellow to red. The degree is based on the color key in the top right of the figure.

The selected genes were then individually overexpressed along with the disruption of *faaA* to confirm that their overexpression would further increase FFA secretory productivity. To overexpress a target gene, the original promoter was replaced by *Ptef1* or *AnPtef1*. As a result, 1–5 positive homokaryon clones for every overexpressed gene were obtained by PCR (Supplementary Figure 2). FFA secretory productivity was quantified using the amount of FFA secreted from one gram of dried cell, and the productivity values were then compared between the Δ*faaA* strain and the derivative overexpressed mutants. The results showed that, in 10 of the 16 overexpressed genes, the FFA secretory productivity was increased by more than two folds (Figure 3A). More specifically, genes of single-subunit enzymes showed a higher yield than genes of subunits of enzyme complexes. Strains overexpressing subunit genes did not show a significantly different yield from that of the Δ*faaA* strain. The overexpressed gene that caused the highest increase in yield was AO090011000863, which encodes a diacylglycerol O-acyltransferase (DGAT), and the overexpressed gene that caused the second highest increase in yield was AO090701000644, which encodes a lipase. FFAs are speculated to be synthesized via triacylglycerol, and the enzymes encoded by these genes are thus a part of the reactions occurring just before FFA production (Figure 4). That is, a diacylglycerol O-acyltransferase produces triacylglycerol from diacylglycerol, and a lipase then produces FFA from triacylglycerol by hydrolysis. These findings corresponded to an improvement of FFA secretory productivity, as expected.

When inspecting the secretory production yield of FFA specifically, the AO090011000863 overexpression mutant also showed the highest yield at 2.33 g/L of culture volume (Figure 3B). In contrast, the AO090701000644 overexpression mutant only showed the 5th highest yield. That is, there were three overexpression mutants showing higher yields than this mutant.

The composition of the FFAs that were secreted by each mutant strain was also analyzed using HPLC (Supplementary Table 11). The results show that the AO090011000863 overexpression mutant had the highest secretory productivity and production yield of FFA. This mutant also had the highest content ratio in free SA, at 32.9%, while it had the lowest content ratio in free LA, at 4.7%. The AO090701000644 overexpression mutant had the second lowest content ratio in free LA, at 7.4%.

## Discussion

Free fatty acids are important in many industries so it is important to be able to manufacture them at a large scale in an environmentally friendly and renewable way. *A. oryzae* can be engineered to produce FFAs and our comprehensive analysis has allowed us to identify genes that can increase FFA secretory productivity in the Δ*faaA* strain. We also focused on some of the upstream and downstream reactions of the FFA biosynthesis step and determined which metabolic functions were affected by the *faaA* disruption that increased FFA productivity.

Detecting differential expression with high confidence in time series data can be difficult owing to factors that are absent in non-time series experiments involving a similar sample size, such as sampling rates and synchronization (Bar-Joseph, 2004; Androulakis et al., 2007). However, the analysis of time series expression was necessary when a clear relationship between gene expression and time was observed in former studies. In our endeavor to select genes for modification, we maximized true positive results using a consensus between multiple algorithms and contrasts by fold-change ranks (Shi et al., 2008) and supplementation by manual verification in the final step. The increase in FFA secretory productivity in the overexpression mutants was mostly as expected, as outlined in another study (Garber et al., 2011; Seyednasrollah et al., 2013). We also introduced a pre- and post-120 h block into the analysis when a clear change in metabolism was evident between those time periods. As shown in a previous study, the pooling of samples to improve dispersion estimation can improve test performance (Oh et al., 2014), particularly in DESeq2. Although pooling was valuable in the identification of genes with the “P” annotation (inorganic ion transport and metabolism) due to being significantly affected by the *faaA* disruption, it was not helpful in selecting genes for modification. Overall, we found that the decision to conduct time series expression analysis is still highly dependent on the individual study. The reason is that the application of multiple algorithms is necessary to account for expected and unexpected factors, such as the strength of the relationship between expression and time and the biological causes underlying detectable differential expression.

The effect of the *faaA* disruption was limited to the increased expression of genes with the COG “P.” This increase was considered reasonable, particularly during the 48–96 h period. In one of the steps of FFA biosynthesis, there is a conversion of palmitoyl-CoA to stearoyl-CoA by an elongase, where cytochrome b5 acts as a cofactor. For cytochrome b5 to function, a ferric ion needs to be chelated to form the heme core of the molecule. Therefore, when *faaA* is disrupted to increase FFA productivity, the processes that facilitate the incorporation of the ferric ion should also increase in a coordinated manner, and this was observed in the gene expression analysis, as expected.

In *A. oryzae*, FFA is considered to be produced from the synthesis of triacylglycerol (i.e., triglyceride) (Figure 4; Tamano et al., 2015). As in most organisms, its metabolic pathway begins with the degradation of glucose to pyruvate by glycolysis. The pyruvate is then transferred to the mitochondria, where it can be further metabolized in the TCA cycle. When there is a sufficient amount of glucose in the cell, this process allows citrate to accumulate in the mitochondria, and some of the excess citrate is secreted into the cytosol. There, the citrate is converted to palmitic acid, which is a C16-saturated FFA, via a four-step biosynthesis reaction. The palmitic acid is then transferred to the endoplasmic reticulum via acylation, where it is subjected to elongation and desaturation processes that produce the various types of fatty acyl-CoA. Triacylglycerol is formed once the fatty acyl-CoA is combined with glycerol and is released to the cytosol as a lipid droplet. When the time comes for *A. oryzae* to recycle triacylglycerol, it is hydrolyzed by lipase to produce FFAs. However, FFAs generated in this way are subject to further degradation by beta-oxidation until finally degraded to acetyl-CoA.

In general, overexpression of genes downstream of palmitic acid biosynthesis contributed more to the increase in FFA production compared to genes upstream, with the exception of AO090005001021. In particular, the overexpression of AO090011000863 and AO090701000644, which are predicted to encode DGAT and lipase, respectively, resulted in the highest two increases in FFA productivity. These two genes are involved in reactions that are immediately upstream of the FFA biosynthesis step in the overall metabolic pathway (Figure 4), suggesting that increased FFA secretory productivity can be achieved by increasing the enzymatic reactions that are closer to FFA biosynthesis steps. This understanding may be utilized in future gene modifications for FFA biosynthesis in *A. oryzae*. This would also be useful information for enhancing the productivity of other metabolites in other microorganisms, by serving as a guide for metabolic engineering. The relationship between FFA productivity and manipulation of the reactions close to the target synthesis step was further confirmed in overexpression of ATP-citrate lyase, acetyl-CoA carboxylase, and FAS genes, which resulted in low or no increase in FFA productivity in this study. This may result from a feedback inhibition effect by fatty acyl-CoA in the case of FAS (Tamano et al., 2013) or the activity of a negative regulator known to be present in *A. oryzae*, SnfA (Kan et al., 2019), in the case of acetyl-CoA carboxylase. For ATP-citrate lyase, it maybe result from a competing metabolic function for the product, acetyl-CoA, as it is also used in the biosynthesis of sterol and other metabolites. Thus, their overexpression seemed to result in no appreciable effects on the overall FFA secretory productivity.

The secretory production yield was the highest for the AO090011000863 overexpression mutant, at 2.33 g/L, whereas it was the fifth highest for the AO090701000644 overexpression mutant, at 2.01 g/L. The fifth rank for the latter is considered to result from the reduced biomass compared to that of the other three overexpression mutants in the second to fourth ranks.

As for the FFA composition, the AO090011000863 overexpression mutant showed the highest content ratio, at 32.9%, in free SA and the lowest content ratio, at 4.7%, in free LA. The mutant also showed the highest content ratio, at 56.1%, in free PA, together with the AO090023000893 overexpression mutant. Therefore, overexpression of AO090011000863 is considered ideal when saturated FFA, such as free PA and free SA, are the target secreted products. In addition, a similar pattern of FFA content ratio was seen to a lesser extent by overexpression of AO090701000644. Hence, the effect on the FFA composition of *A. oryzae* cells by the overexpression of these genes was considered similar. In contrast, when free LA, a desaturated FFA, is the targeted secreted product, overexpression of either AO090011000488 or AO090001000224 is considered most important, because their overexpression mutants showed content ratios of free LA at more than 20% as well as relatively high secretory productivities of FFA. Both genes are predicted to encode desaturases, and it is thus considered reasonable to see such characteristics resulting from their overexpression.

## Data Availability Statement

The datasets supporting the results of this study are included in the article/Supplementary Material. The *Aspergillus* oryzae RIB40 ASM18445v3 reference genome datasets analyzed in this study are publicly available in the NCBI GenBank database (accession No. PRJNA20809). The RNA-seq raw data generated in this study using an Illumina HiSeq 2500 for all samples have been deposited in the NCBI GenBank database and is publicly available (accession No. PRJDB8293).

## Author Contributions

KT conceived and conducted the experiments. PSW analyzed the results. SA supervised the project. All authors reviewed the manuscript.

## Conflict of Interest

The authors declare that the research was conducted in the absence of any commercial or financial relationships that could be construed as a potential conflict of interest.

## Footnotes

1 www.ncbi.nlm.nih.gov/

2 https://nribf21.nrib.go.jp/CAoGD/
