# Supplementary material for: Improvement of Free Fatty Acid Secretory Productivity in Aspergillus oryzae by Comprehensive Analysis on Time-Series Gene Expression
Source: Front Microbiol. 2021 Apr 9;12:605095. doi: 10.3389/fmicb.2021.605095 (PMC8062725; doi:10.3389/fmicb.2021.605095)
Supplement: Supplementary file 1 [file Data_Sheet_1.PDF]

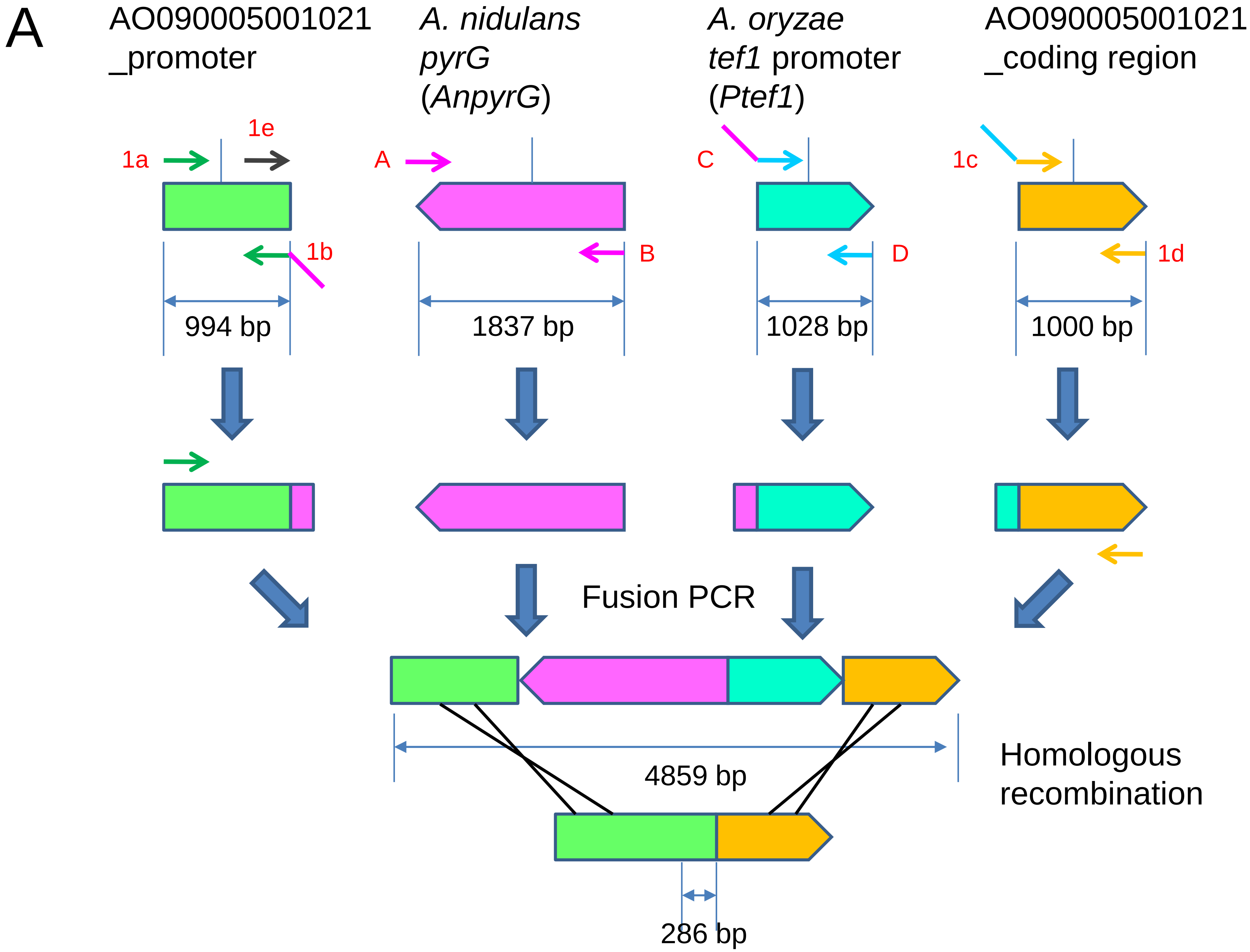

FIG. S1A. Construction of the DNA fragment for overexpressing AO090005001021 in *A. oryzae faaA* disruptant. The 4859 bp long DNA fragment was constructed for the overexpression. Primers used for the construction and clone check are shown as colored and black arrows, respectively.

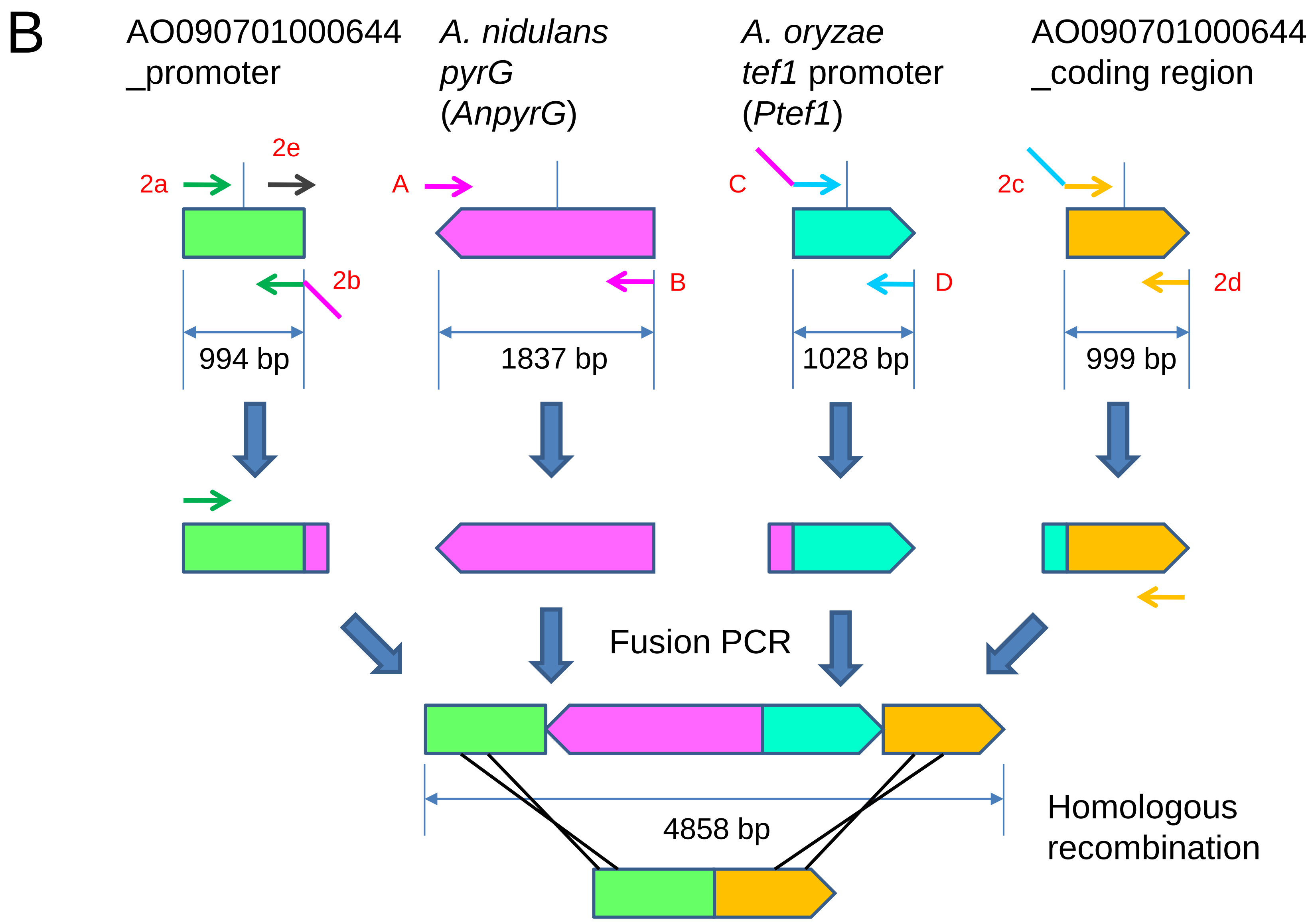

FIG. S1B. Construction of the DNA fragment for overexpressing AO090701000644 in *A. oryzae faaA* disruptant. The 4858 bp long DNA fragment was constructed for the overexpression. Primers used for the construction and clone check are shown as colored and black arrows, respectively.

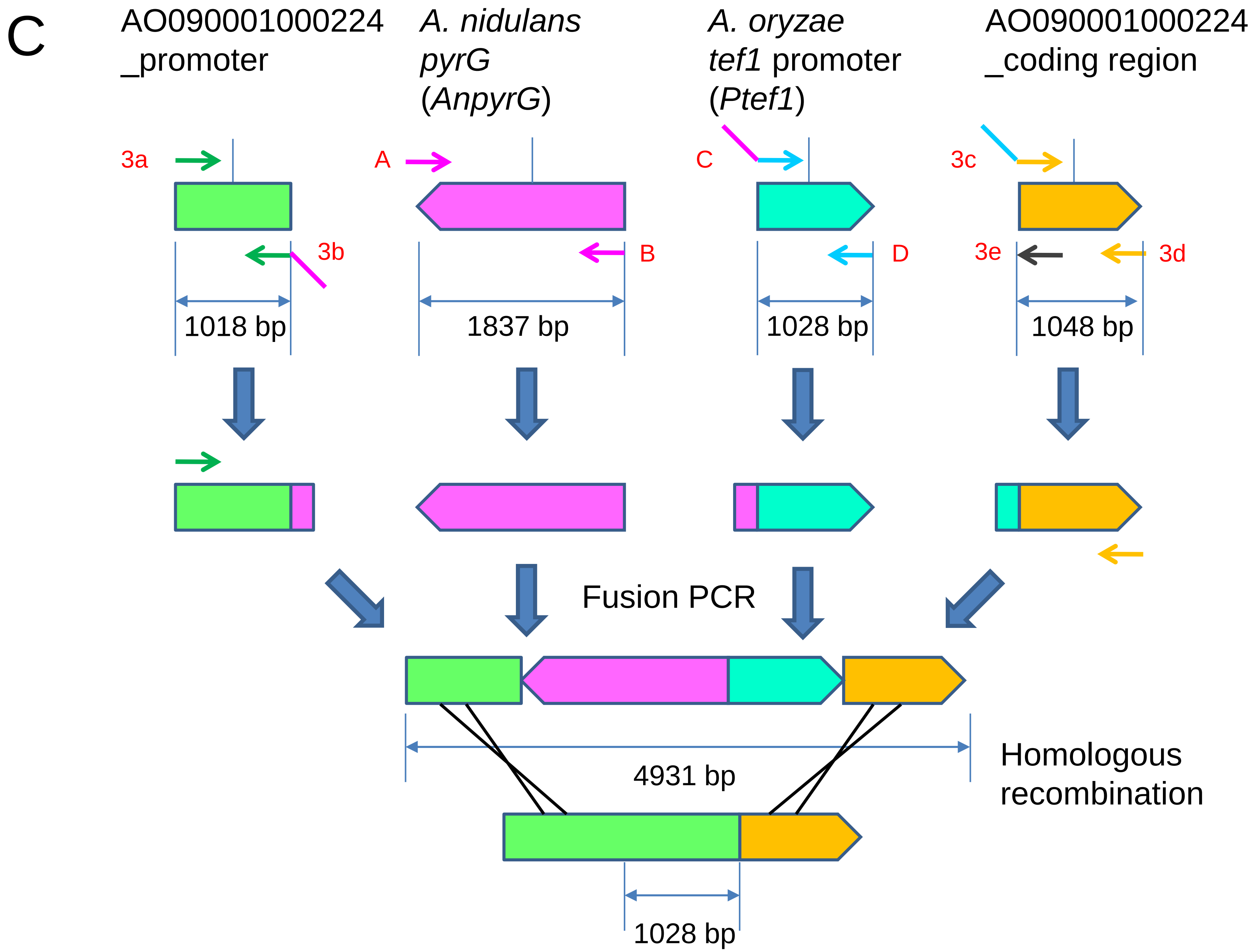

FIG. S1C. Construction of the DNA fragment for overexpressing AO090001000224 in *A. oryzae faaA* disruptant. The 4931 bp long DNA fragment was constructed for the overexpression. Primers used for the construction and clone check are shown as colored and black arrows, respectively.

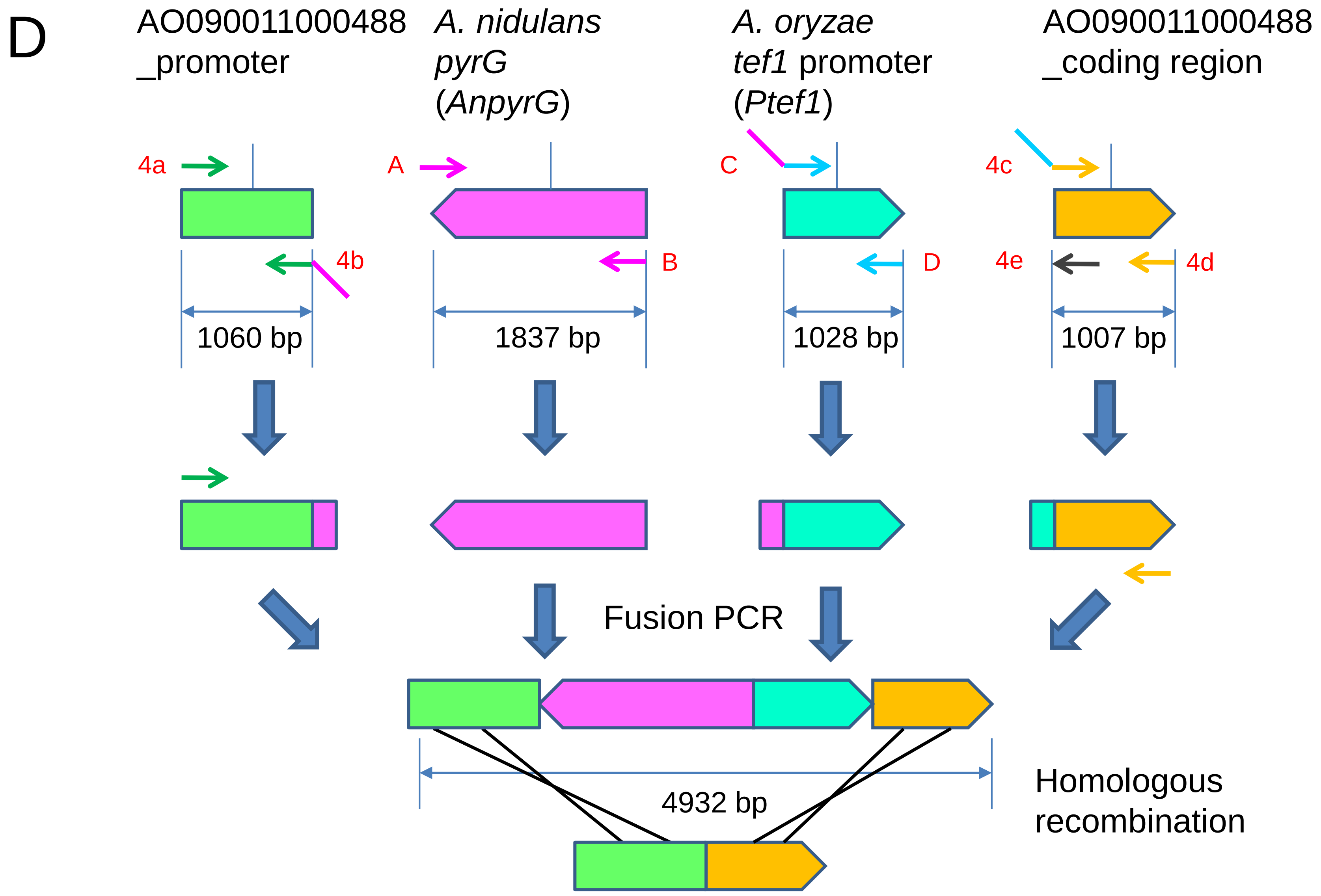

FIG. S1D. Construction of the DNA fragment for overexpressing AO090011000488 in *A. oryzae faaA* disruptant. The 4932 bp long DNA fragment was constructed for the overexpression. Primers used for the construction and clone check are shown as colored and black arrows, respectively.

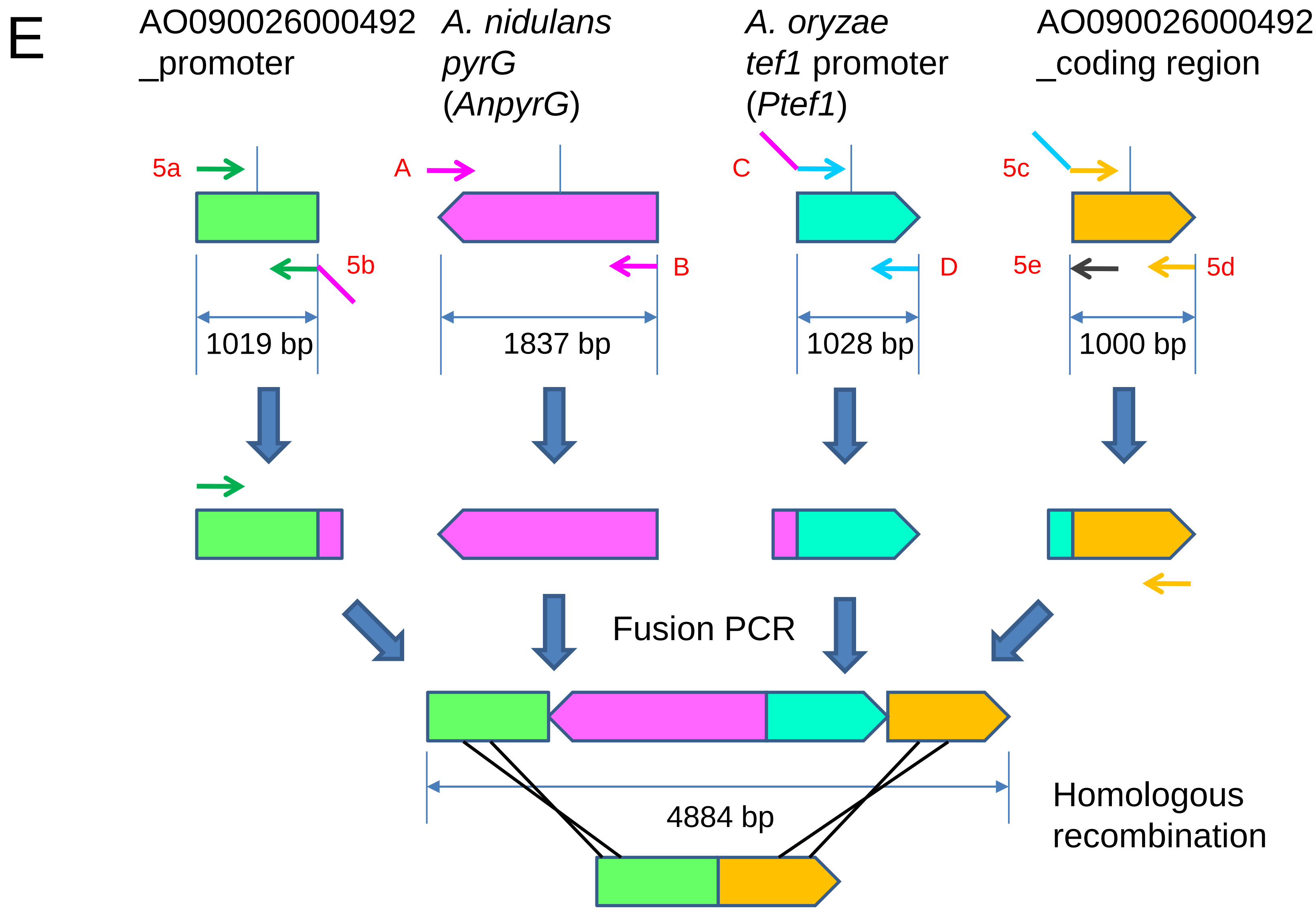

FIG. S1E. Construction of the DNA fragment for overexpressing AO090026000492 in *A. oryzae faaA* disruptant. The 4884 bp long DNA fragment was constructed for the overexpression. Primers used for the construction and clone check are shown as colored and black arrows, respectively.

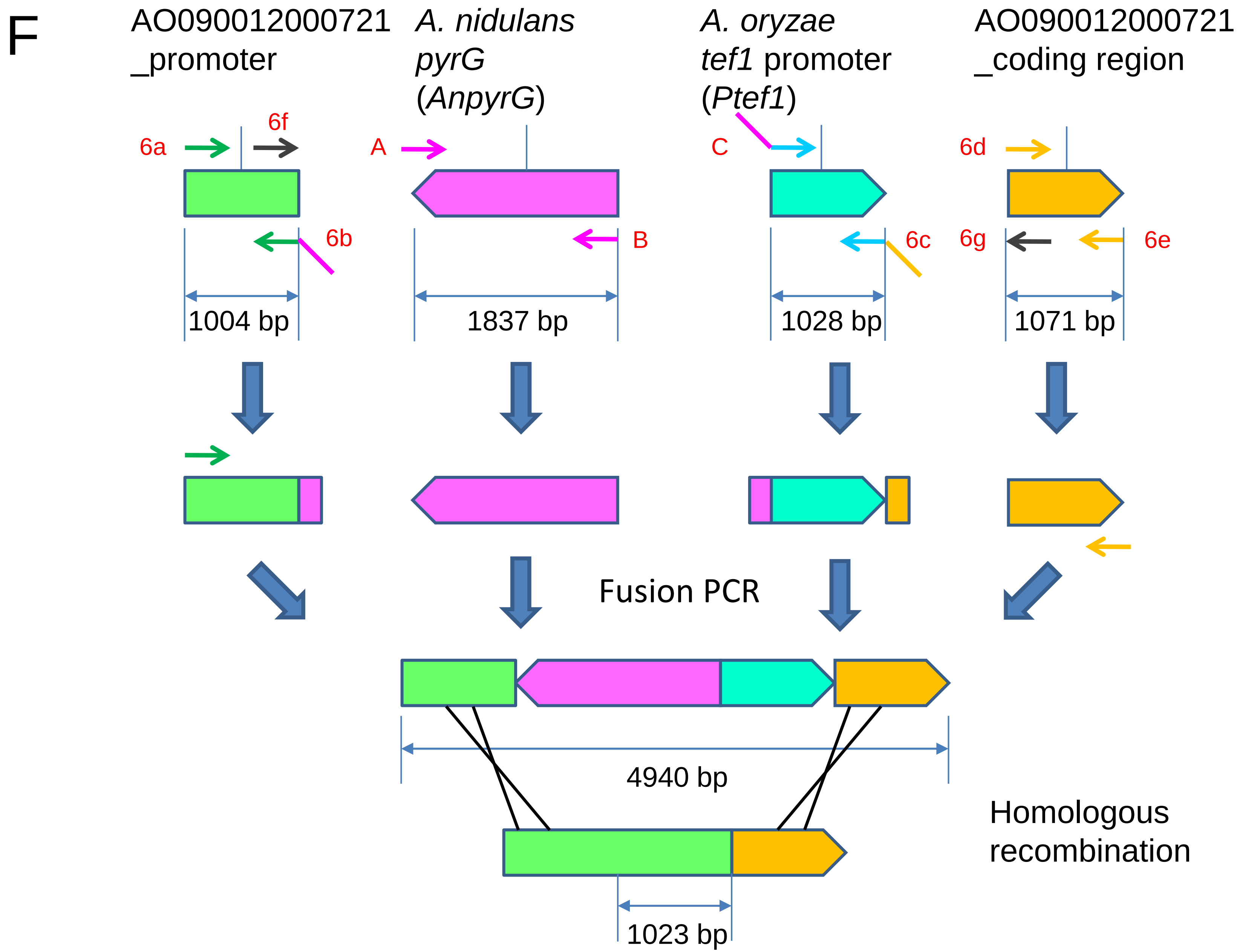

FIG. S1F. Construction of the DNA fragment for overexpressing AO090012000721 in *A. oryzae faaA* disruptant. The 4940 bp long DNA fragment was constructed for the overexpression. Primers used for the construction and clone check are shown as colored and black arrows, respectively.

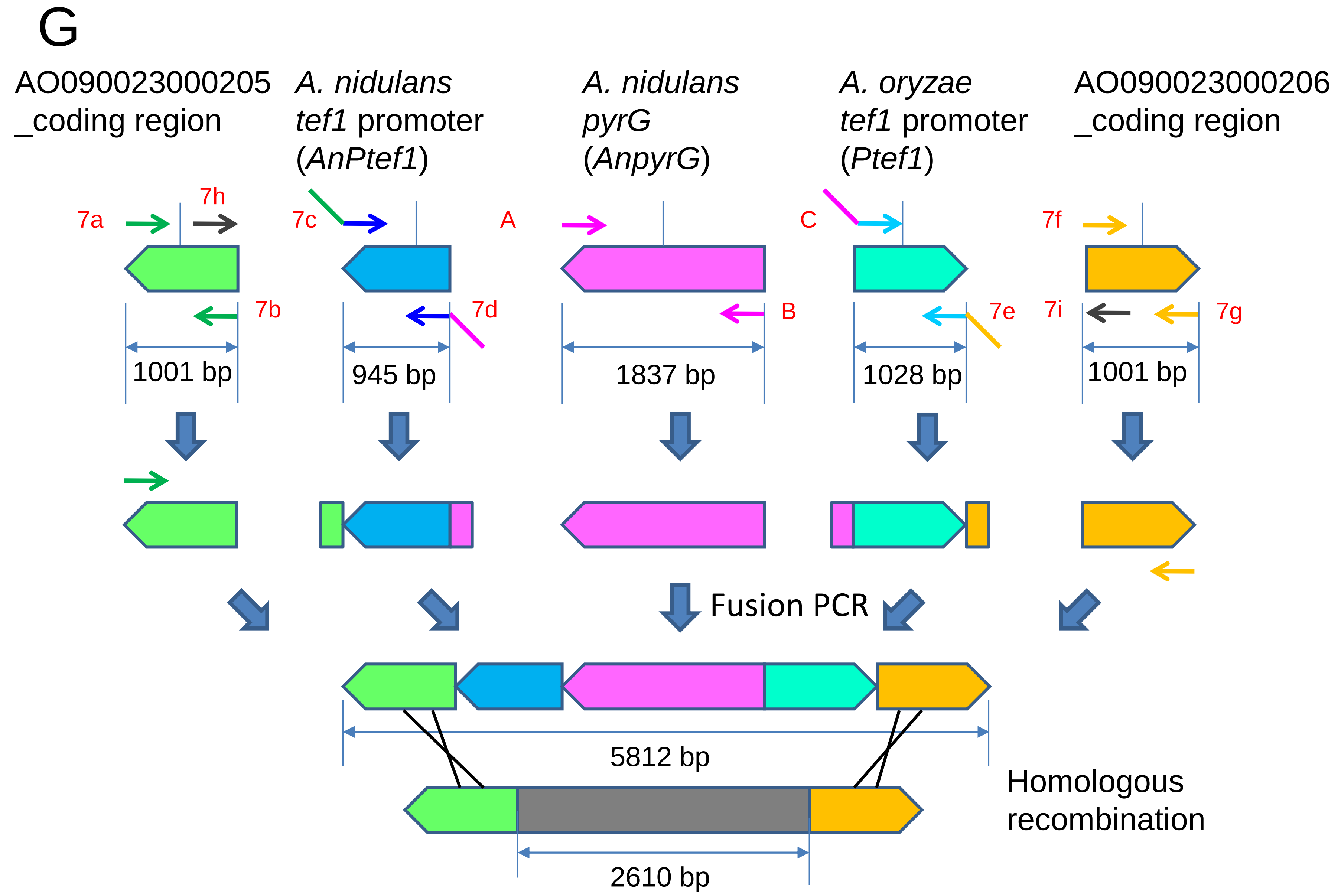

FIG. S1G. Construction of the DNA fragment for overexpressing AO090023000205 (ATP-citrate lyase subunit 1) and AO090023000206 (ATP-citrate lyase subunit 2) in *A. oryzae faaA* disruptant. The 5812 bp long DNA fragment was constructed for the overexpression. Primers used for the construction and clone check are shown as colored and black arrows, respectively.

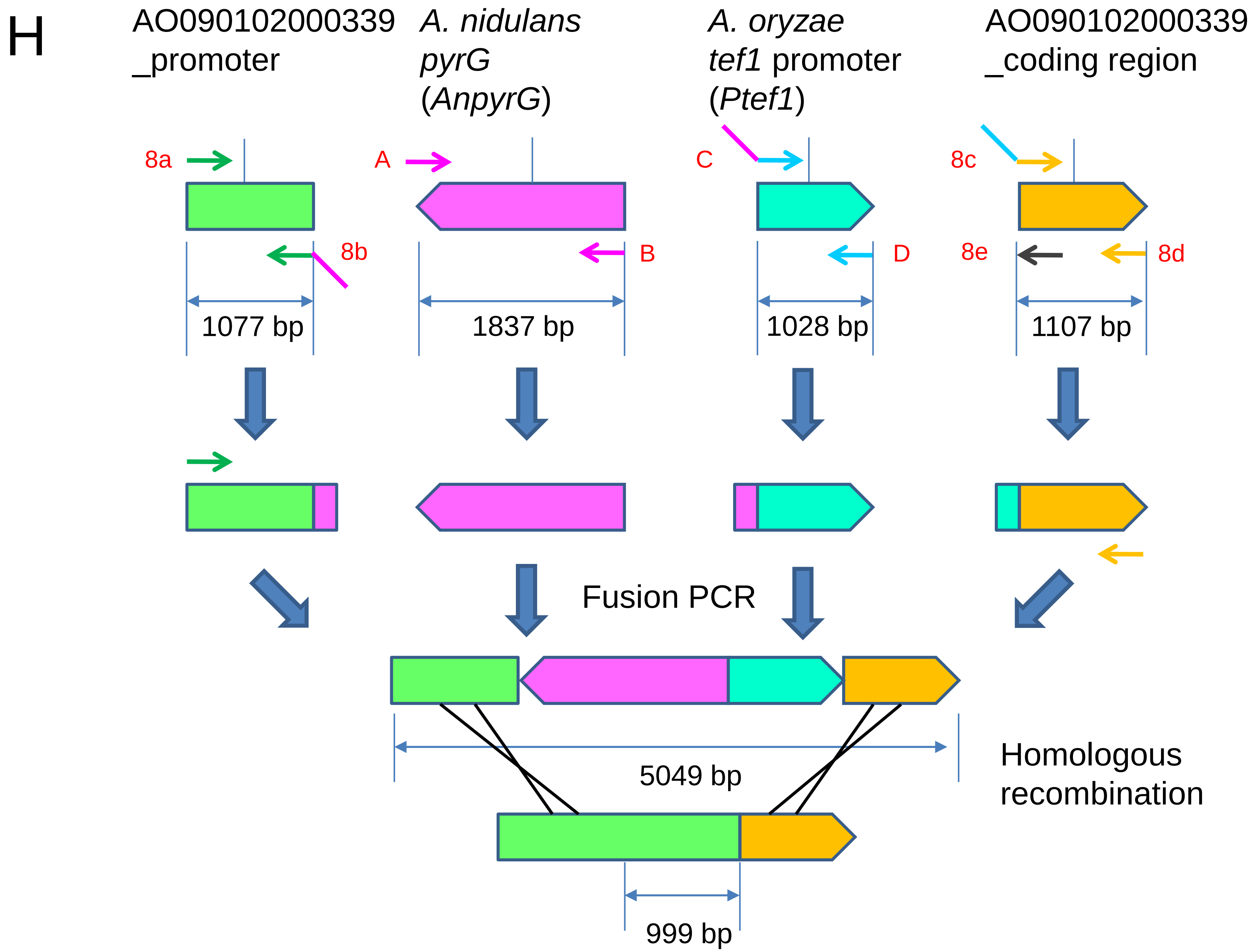

FIG. S1H. Construction of the DNA fragment for overexpressing AO090102000339 in *A. oryzae faaA* disruptant. The 5049 bp long DNA fragment was constructed for the overexpression. Primers used for the construction and clone check are shown as colored and black arrows, respectively.

I

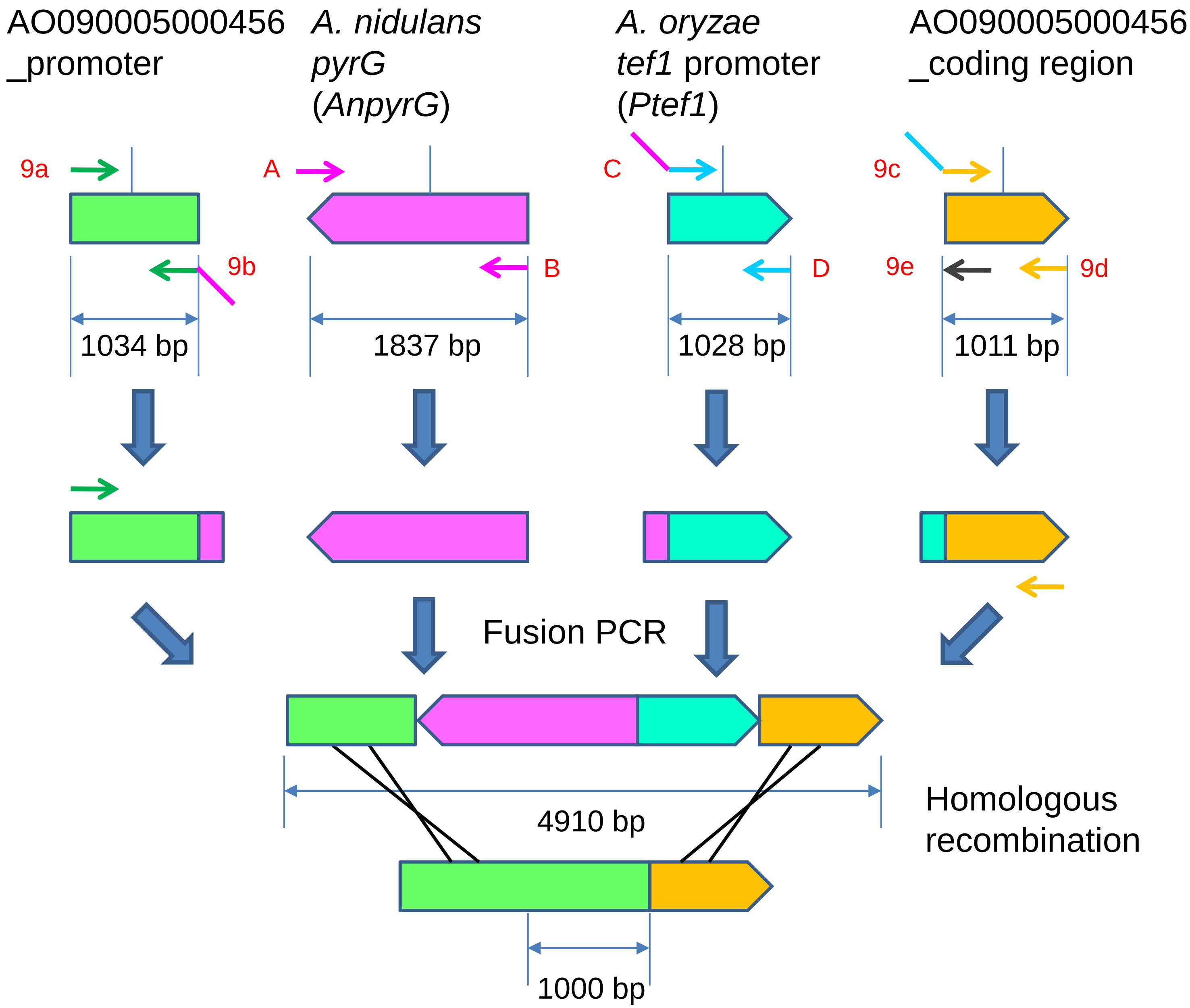

FIG. S1I. Construction of the DNA fragment for overexpressing AO090005000456 in *A. oryzae faaA* disruptant. The 4910 bp long DNA fragment was constructed for the overexpression. Primers used for the construction and clone check are shown as colored and black arrows, respectively.

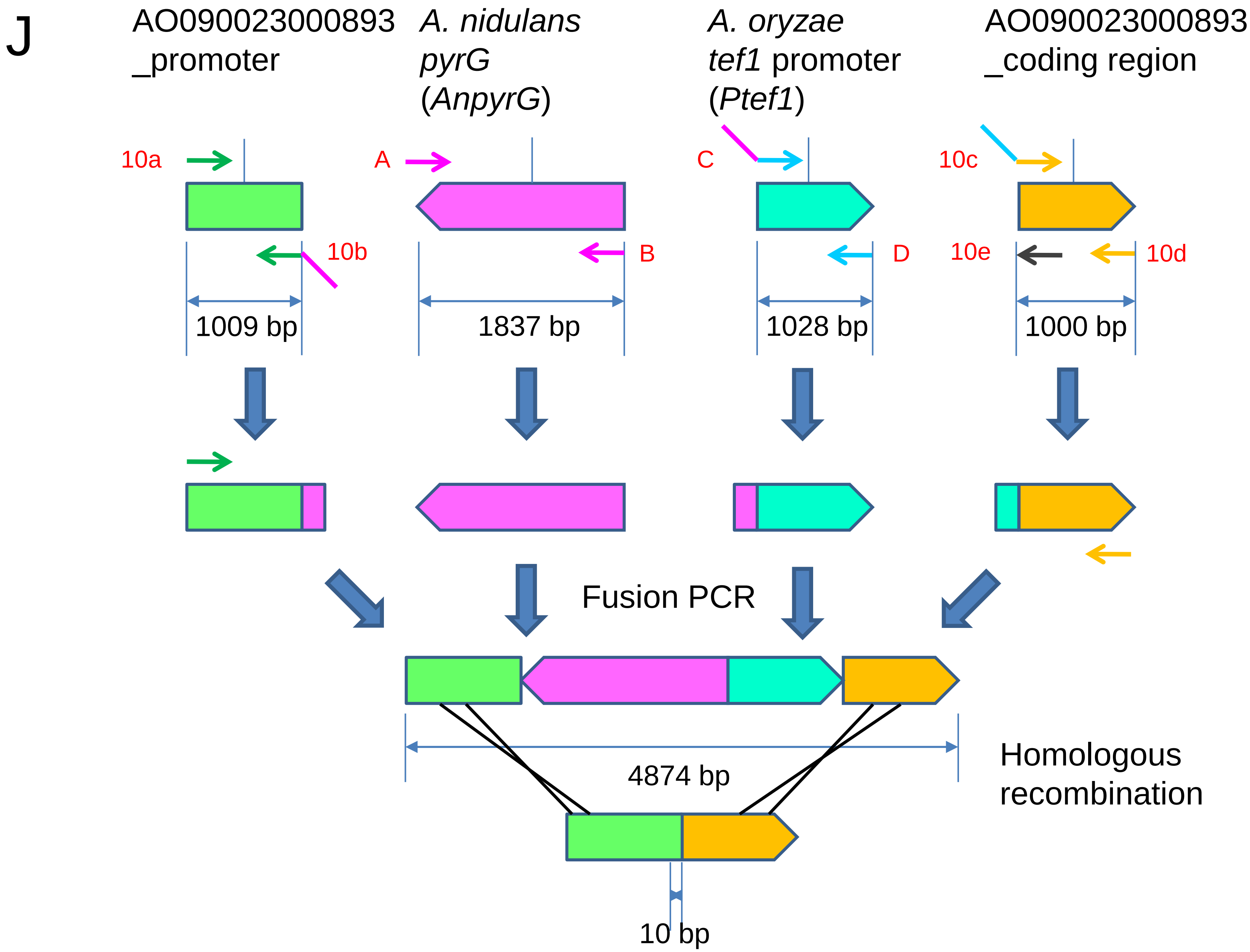

FIG. S1J. Construction of the DNA fragment for overexpressing AO090023000893 in *A. oryzae faaA* disruptant. The 4874 bp long DNA fragment was constructed for the overexpression. Primers used for the construction and clone check are shown as colored and black arrows, respectively.

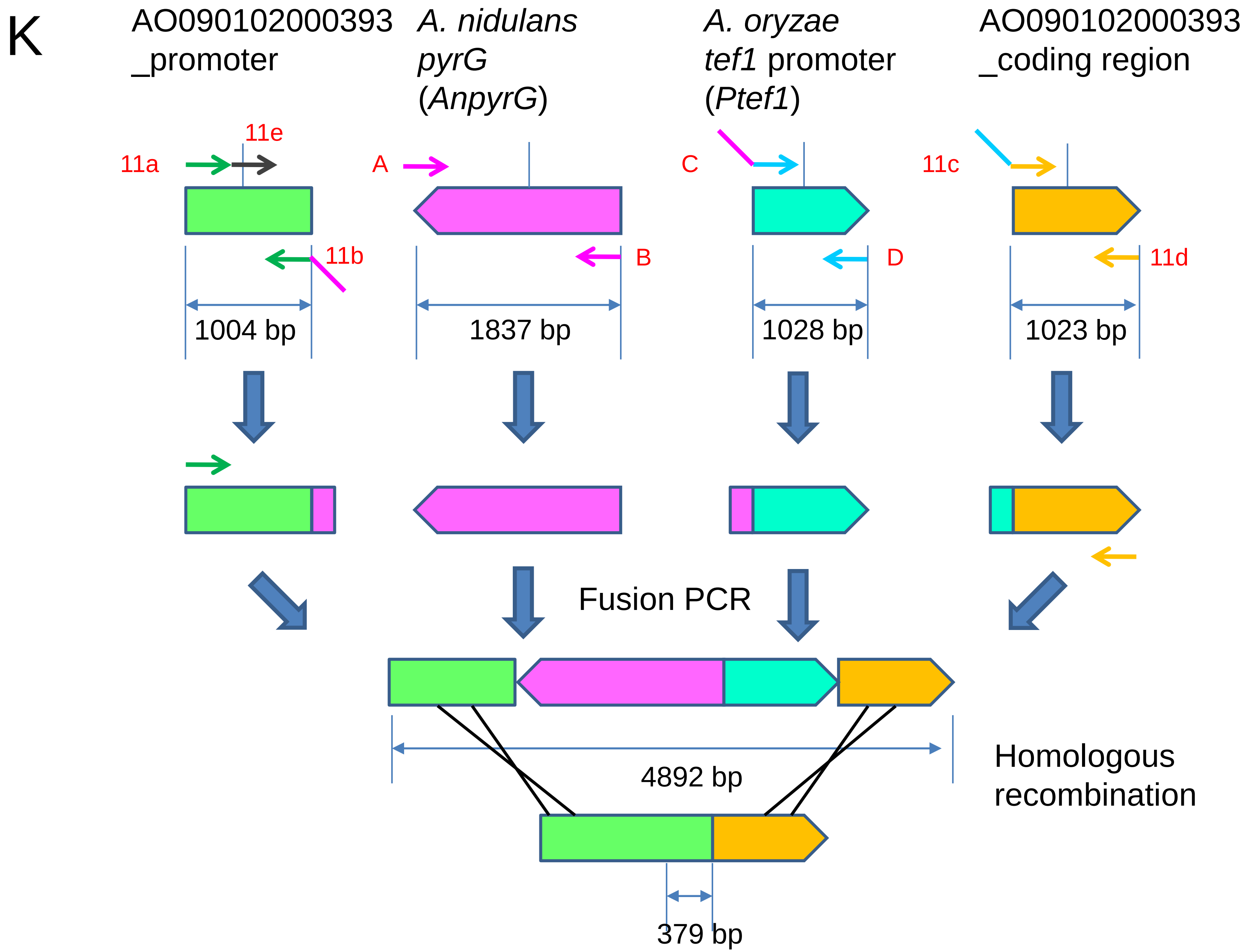

FIG. S1K. Construction of the DNA fragment for overexpressing AO090102000393 in *A. oryzae faaA* disruptant. The 4892 bp long DNA fragment was constructed for the overexpression. Primers used for the construction and clone check are shown as colored and black arrows, respectively.

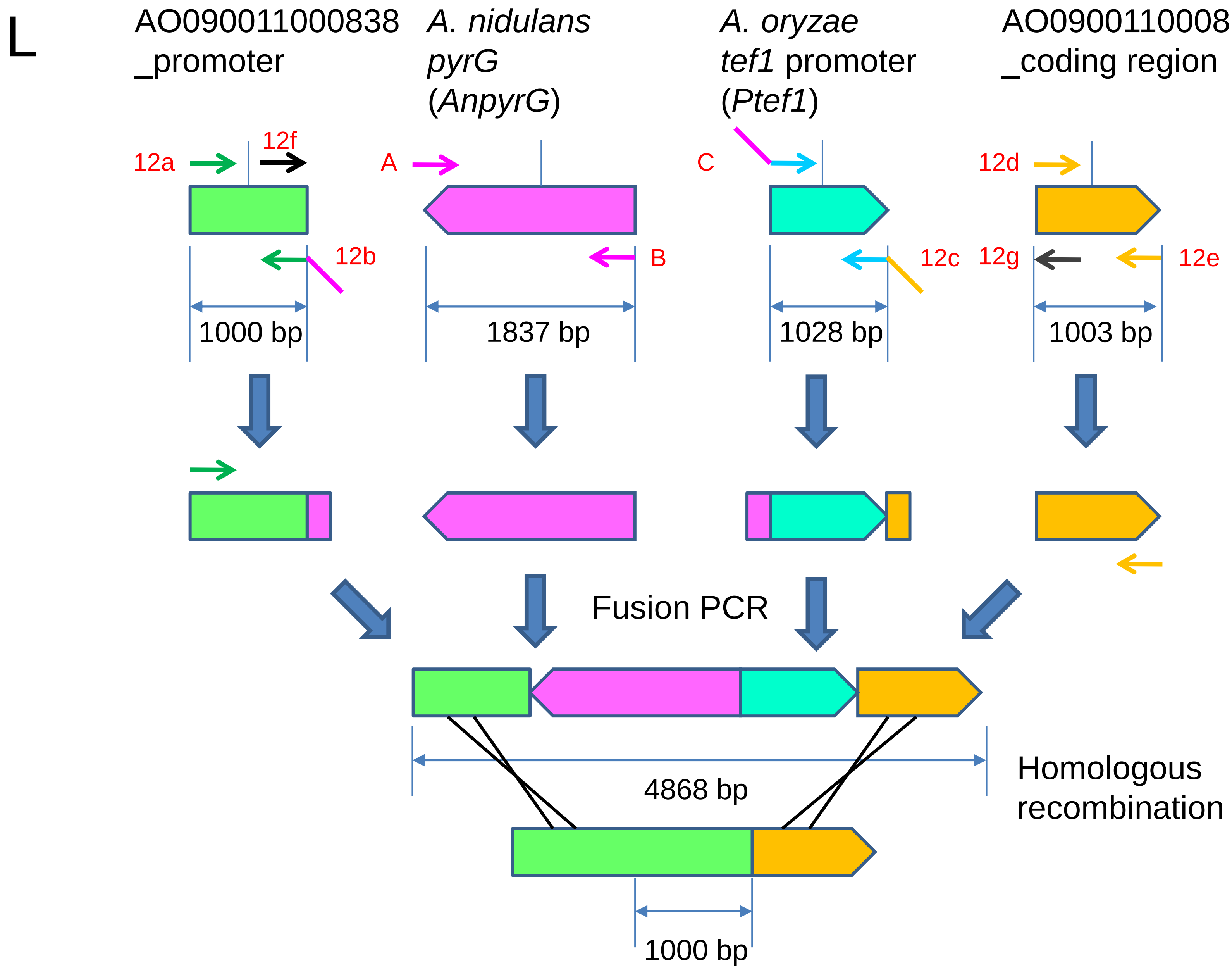

FIG. S1L. Construction of the DNA fragment for overexpressing AO090011000838 in *A. oryzae faaA* disruptant. The 4868 bp long DNA fragment was constructed for the overexpression. Primers used for the construction and clone check are shown as colored and black arrows, respectively.

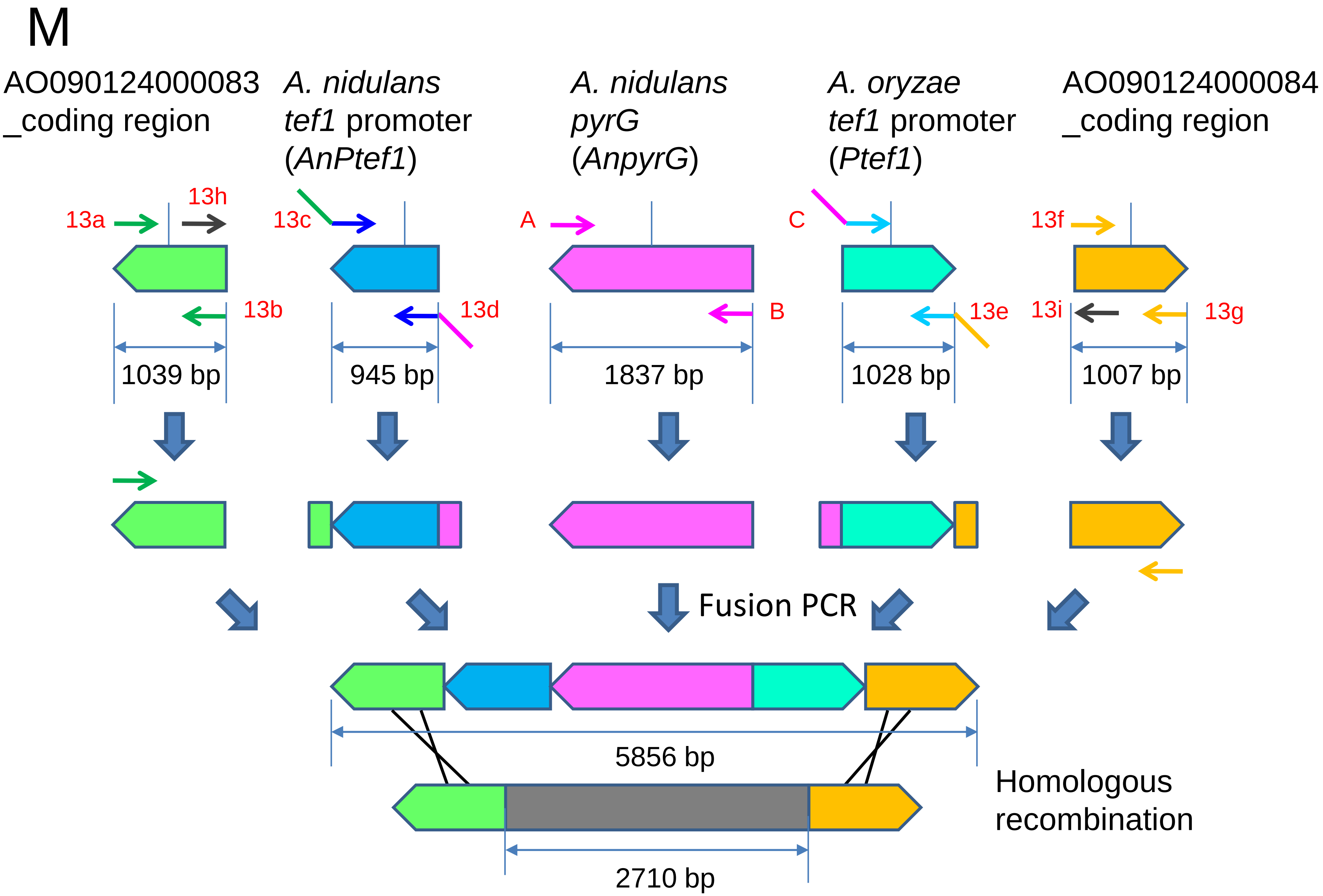

FIG. S1M. Construction of the DNA fragment for overexpressing AO090124000083 (Fatty acid synthase subunit 1) and AO090124000084 (Fatty acid synthase subunit 2) in *A. oryzae faaA* disruptant. The 5856 bp long DNA fragment was constructed for the overexpression. Primers used for the construction and clone check are shown as colored and black arrows, respectively.

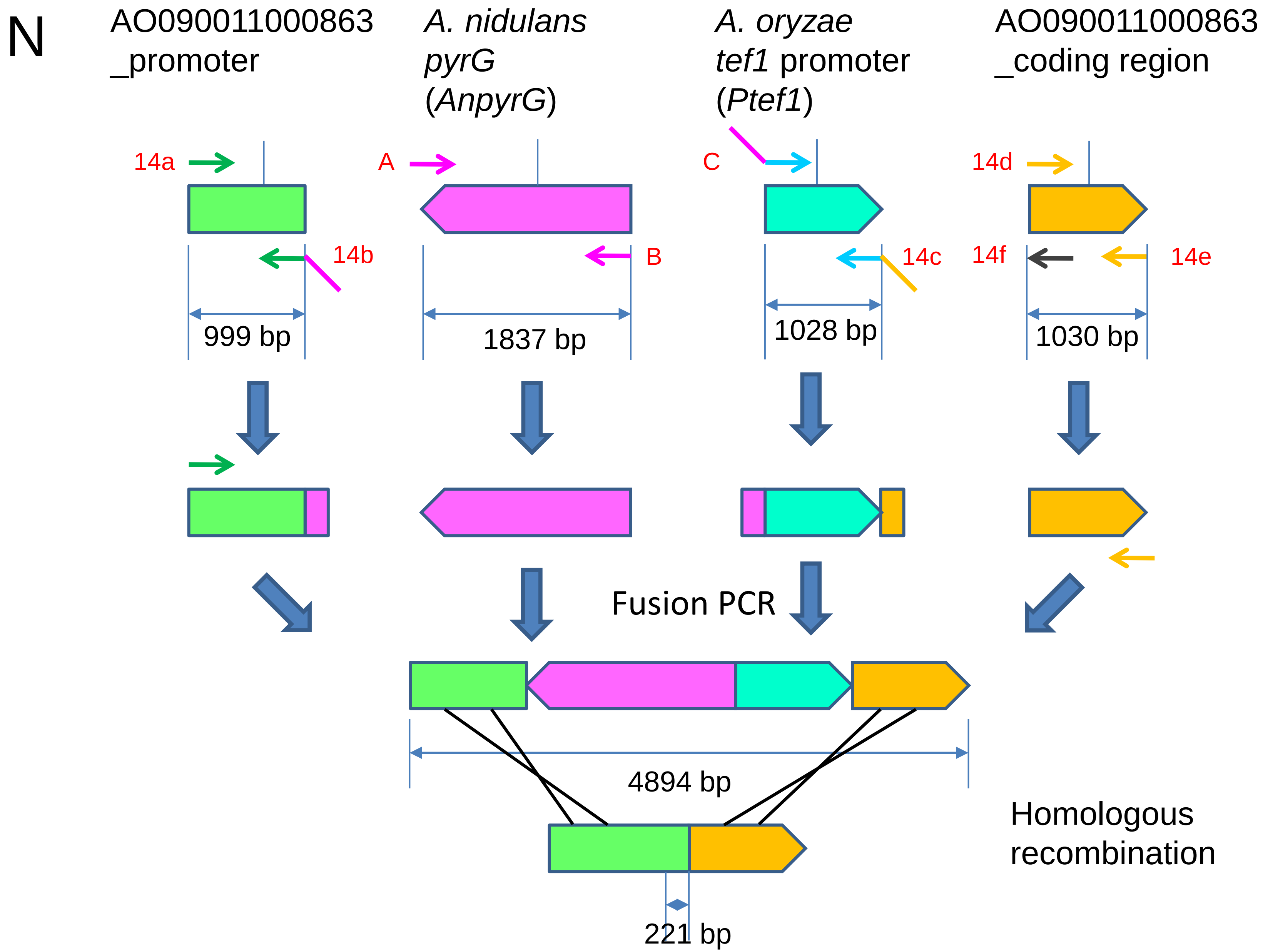

FIG. S1N. Construction of the DNA fragment for overexpressing AO090011000863 in *A. oryzae faaA* disruptant. The 4894 bp long DNA fragment was constructed for the overexpression. Primers used for the construction and clone check are shown as colored and black arrows, respectively.
