# Supplementary material for: Improvement of Free Fatty Acid Secretory Productivity in Aspergillus oryzae by Comprehensive Analysis on Time-Series Gene Expression
Source: Front Microbiol. 2021 Apr 9;12:605095. doi: 10.3389/fmicb.2021.605095 (PMC8062725; doi:10.3389/fmicb.2021.605095)
Supplement: Supplementary file 2 [file Data_Sheet_2.PDF]

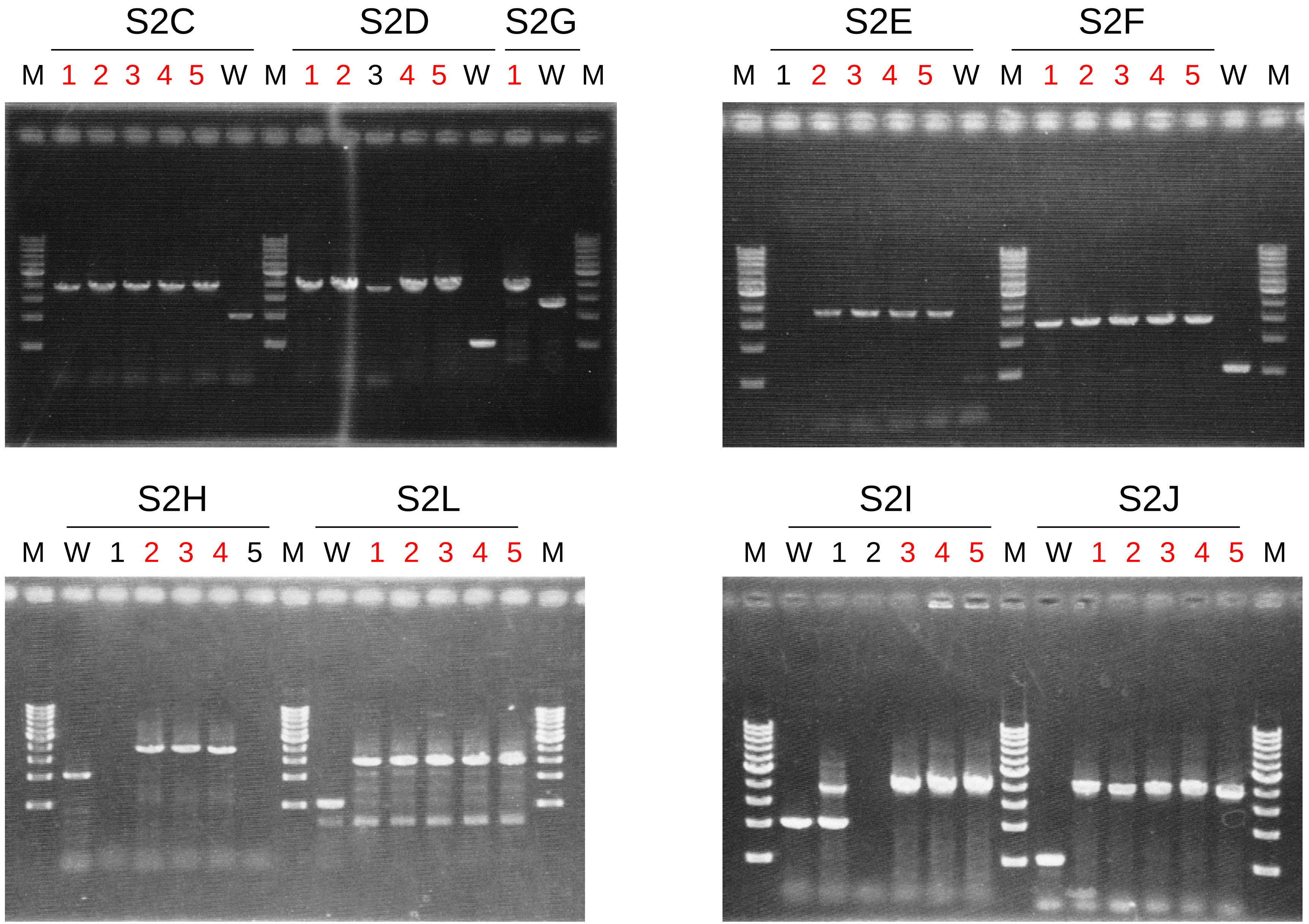

FIG. S2. Clone check of the overexpression mutants isolated after transformation. Clones for overexpressing AO090001000224 (S2C), AO090011000448 (S2D), AO090023000205&6 (S2G), AO090026000492 (S2E), AO090012000721 (S2F), AO090102000339 (S2H), AO090011000838 (S2L), AO090005000456 (S2I), and AO090023000893 (S2J) are shown. Lane M: 1 kb DNA ladder marker [1-10 kb], lane W: Wild-type strain as a negative control. Clones that were considered to be positive homokaryons are shown in red.

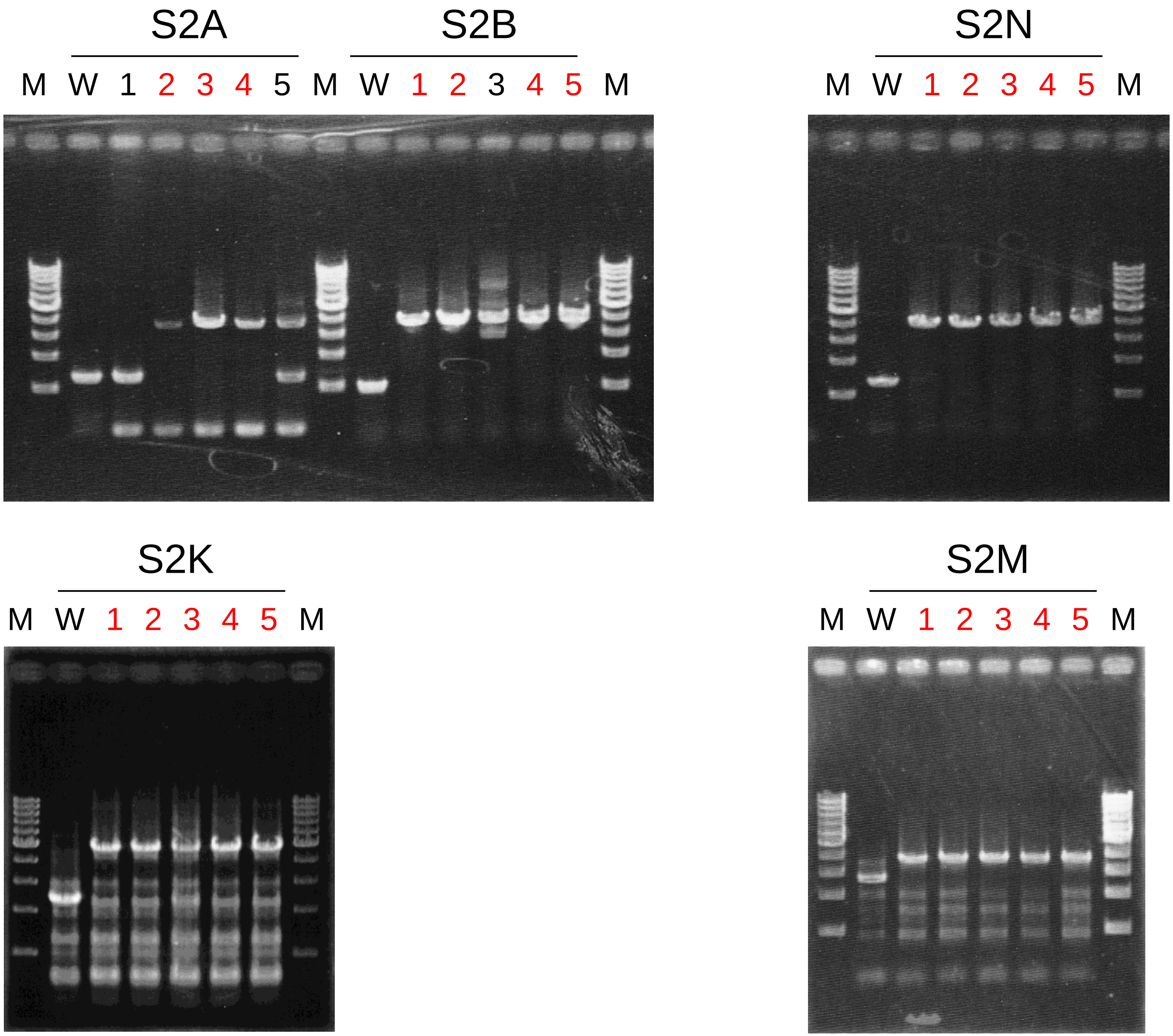

FIG. S2 (continued). Clone check of the overexpression mutants isolated after transformation. Clones for overexpressing AO090005001021 (S2A), AO090701000644 (S2B), AO090011000863 (S2N), AO090102000393 (S2K), and AO090124000083&4 (S2M) are shown. Lane M: 1 kb DNA ladder marker [1-10 kb], lane W: Wild-type strain as a negative control. Clones that were considered to be positive homokaryons are shown in red.
