# Supplementary material for: Improvement of Free Fatty Acid Secretory Productivity in Aspergillus oryzae by Comprehensive Analysis on Time-Series Gene Expression
Source: Front Microbiol. 2021 Apr 9;12:605095. doi: 10.3389/fmicb.2021.605095 (PMC8062725; doi:10.3389/fmicb.2021.605095)
Supplement: Supplementary file 4 [file Table_2.DOCX]

**Supplementary Table S2.** The list of DNA primers used in this study.

| No. | Name | Sequence (5' to 3') |
| --- | --- | --- |
| A | PU_AnpyrG | AGCATATCGTTCAGAGCTGG |
| B | PL_AnpyrG | ACTAGCCAGCTAGCTCAGTC |
| C | RU_AoPtef* | gtaagactgagctagctggctagtGTAGATTGTCACCGCTCAGG |
| D | RL_AoPtef | TTTGAAGGTGGTGCGAACTTT |
| 1a | 005-1021_LU | AACATATCCCTCGGCCCTGCTTTG |
| 1b | 005-1021_LL* | gtgaccagctctgaacgatatgctGGCAATGAGGCAATTGACGA |
| 1c | 005-1021_RU* | tacaaagttcgcaccaccttcaaaATGTTCGATCTGAACCTCG |
| 1d | 005-1021_RL | CTTAGACTGGGGAGGAAGGGTGTA |
| 1e | 005-1021_cU | TTCGTCAATTGCCTCATTGC |
| 2a | 701-0644_LU | GAGGGAAATTCTGGACGATTCTCG |
| 2b | 701-0644_LL* | gtgaccagctctgaacgatatgctCTTTGCCAGTGTGCTTCGCA |
| 2c | 701-0644_RU* | tacaaagttcgcaccaccttcaaaATGCGCTTCCTCTCCGGCTT |
| 2d | 701-0644_RL | TTGCAGGCATCGGCGTGGATAAAG |
| 2e | 701-0644_cU | TGCGAAGCACACTGGCAAAG |
| 3a | 001-0224_LU | GGTATGTAGTCTGGGTATGTGCTG |
| 3b | 001-0224_LL* | gtgaccagctctgaacgatatgctACGTCGTTCCAAGTGTTTGC |
| 3c | 001-0224_RU* | tacaaagttcgcaccaccttcaaaATGTCTTCCACAGCTATCCC |
| 3d | 001-0224_RL | TGGCATCGTTAGCACATGTCTCGA |
| 3e | 001-0224_cL | GGGATAGCTGTGGAAGACAT |
| 4a | 011-0488_LU | CCGGGTTCTACTGGAGAGTTAGTT |
| 4b | 011-0488_LL* | gtgaccagctctgaacgatatgctGATATGTAGAGAACGATCGTAT |
| 4c | 011-0488_RU* | tacaaagttcgcaccaccttcaaaATGGCCGATACTCTTAAAGC |
| 4d | 011-0488_RL | GGTGCATTCGTCACAATATGGTGG |
| 4e | 011-0488_cL | GCTTTAAGAGTATCGGCCAT |
| 5a | 026-0492_LU | GGACGGATAAGATATCACAGAGTACG |
| 5b | 026-0492_LL* | gtgaccagctctgaacgatatgctGATTGGCGCCTTTTCCCCTT |
| 5c | 026-0492_RU* | tacaaagttcgcaccaccttcaaaATGGATTTCCTATCGAAGCA |
| 5d | 026-0492_RL | ATGGTTCCGGCGATACAGGTCAAG |
| 5e | 026-0492_cL | TGCTTCGATAGGAAATCCAT |
| 6a | 012-0721_LU | TGCGTTATTGCTTGATCGACTAGC |
| 6b | 012-0721_LL* | gtgaccagctctgaacgatatgctAGGAACCTCAGGCTATGTTA |
| 6c | 012-0721_RL(Ao_tef)* | agctagaagcgcgagggtgcgcatTTTGAAGGTGGTGCGAACTT |
| 6d | 012-0721_RU | ATGCGCACCCTCGCGCTTCT |
| 6e | 012-0721_RL | CCTAATCCAACGGACCCAATTCCA |
|  | **Supplementary Table S1** (continued) | |
| 6f | 012-0721_cU | TAACATAGCCTGAGGTTCCT |
| 6g | 012-0721_cL | AGAAGCGCGAGGGTGCGCAT |
| 7a | 023-0205_LU | AGGCACCGGTCTTGGCATCCAACT |
| 7b | 023-0205_LL | ATGTCCGCCAAGTCGATTCT |
| 7c | 023-0205_LU(An_tef)* | ctcgagaatcgacttggcggacatGGTGAAGGTTGTGTTATGTT |
| 7d | 023-0205_LLn(An_tef)p* | gtgaccagctctgaacgatatgctTGGCATATAGTACGCCTGAA |
| 7e | 023-0206_RL(Ao_tef)* | gacaagaggagcggtagaagacatTTTGAAGGTGGTGCGAACTT |
| 7f | 023-0206_RU | ATGTCTTCTACCGCTCCTCT |
| 7g | 023-0206_RL | CGTCTTGAACATGCTGGCGCAAGT |
| 7h | 023-0205_cU | AGAATCGACTTGGCGGACAT |
| 7i | 023-0206_cL | ACAAGAGGAGCGGTAGAAGA |
| 8a | 102-0339_LU | CACTACCAGCTGCCACCGAATTGA |
| 8b | 102-0339_LL* | gtgaccagctctgaacgatatgctTCAGAATCCTTGCGAACCTG |
| 8c | 102-0339_RU* | tacaaagttcgcaccaccttcaaaATGGCGCTCGATGCAAAATC |
| 8d | 102-0339_RL | ACGAACTCGTCCCAGTCCATTACG |
| 8e | 102-0339_cL | AAGTAGCAACTGTCGGTGAT |
| 9a | 005-0456_LU | GCCTGCTTATCAGTGACCATAACG |
| 9b | 005-0456_LL* | gtgaccagctctgaacgatatgctTTCTTGCCACTTCGTGGTGG |
| 9c | 005-0456_RU* | tacaaagttcgcaccaccttcaaaATGTCTGCAAAGACGGCAGA |
| 9d | 005-0456_RL | TCTTCTTCTGGAGCTGCTGAACAC |
| 9e | 005-0456_cL | TCTGCCGTCTTTGCAGACAT |
| 10a | 023-0893_LU | CCCGTGAAGTGTATTAGGAACCTC |
| 10b | 023-0893_LL* | gtgaccagctctgaacgatatgctCGGGATAGGAGAAGGAAAGA |
| 10c | 023-0893_RU* | tacaaagttcgcaccaccttcaaaATGCCCTCCGCCGTCGT |
| 10d | 023-0893_RL | GTGGATTTCGCCTTCTGCTCTGTG |
| 10e | 023-0893_cL | GACTATCGGTGGTCACGA |
| 11a | 102-0393_LU | GCTCGATATATTTCTGAGAGCTCAGG |
| 11b | 102-0393_LL* | gtgaccagctctgaacgatatgctTTTTGGTTTCGCCGGCACGT |
| 11c | 102-0393_RU* | tacaaagttcgcaccaccttcaaaATGGCGCTCGAATTCCTACA |
| 11d | 102-0393_RL | TAACAGCCTGTCTGCCAGTGTTAC |
| 11e | 102-0393_LU2 | AACTCTTTGTCCATGCCAAC |
| 12a | 011-0838_LU | ATAAGAGGCTCCGAAGACCGAACT |
| 12b | 011-0838_LL* | gtgaccagctctgaacgatatgctGTATCGTGATTACTCTTGCC |
| 12c | 011-0838_RL(Ao_tef)* | cgaggttccgttgacgtgggccatTTTGAAGGTGGTGCGAACTT |
| 12d | 011-0838_RU | ATGGCCCACGTCAACGGAAC |
|  | **Supplementary Table S1** (continued) | |
| 12e | 011-0838_RL | ATCGGCCAGCAGCTGAACTTCCAA |
| 12f | 011-0838_cU | GGCAAGAGTAATCACGATAC |
| 12g | 011-0838_cL | GTTCCGTTGACGTGGGCCAT |
| 13a | 124-0083_LU | CCTTGGTCAGAGCATCAATTGCTG |
| 13b | 124-0083_LL | ATGCGTCCTGAAATCGAACA |
| 13c | 124-0083_LU(An_tef)* | ctcctgttcgatttcaggacgcatGGTGAAGGTTGTGTTATGTT |
| 13d | 023-0205_LLn(An_tef)p* | gtgaccagctctgaacgatatgctTGGCATATAGTACGCCTGAA |
| 13e | 124-0084_RL(Ao_tef)* | agggcctgtagatgttccatacatTTTGAAGGTGGTGCGAACTT |
| 13f | 124-0084_RU | ATGTATGGAACATCTACAGG |
| 13g | 124-0084_RL | AACACCGATGGAGCAATCGATGTG |
| 13h | 124-0083_cU | TGTTCGATTTCAGGACGCAT |
| 13i | 124-0084_cL | CCTGTAGATGTTCCATACAT |
| 14a | 011-0863_LU | CAGTGACAAGCCCTGTTGAATTGG |
| 14b | 011-0863_LL* | gtgaccagctctgaacgatatgctCCGAAGGAGGTGAAGTTCTG |
| 14c | 011-0863_RL(Ao_tef)* | ttcaataatcaatcggagattcatTTTGAAGGTGGTGCGAACTT |
| 14d | 011-0863_RU | ATGAATCTCCGATTGATTAT |
| 14e | 011-0863_RL | GCTTGCCGTCAAAGGACTATATCC |
| 14f | 011-0863_cL | CGATAAAACGTTCAGCTCTG |

*Tail of the primer is shown in lower case.
